# Transcriptome analysis of porcine PBMCs after in vitro stimulation by LPS or PMA/ionomycin using an expression array targeting the pig immune response

**DOI:** 10.1186/1471-2164-11-292

**Published:** 2010-05-11

**Authors:** Yu Gao, Laurence Flori, Jérome Lecardonnel, Diane Esquerré, Zhi-Liang Hu, Angélique Teillaud, Gaëtan Lemonnier, Francois Lefèvre, Isabelle P Oswald, Claire Rogel-Gaillard

**Affiliations:** 1 INRA, UMR 1313 de Génétique Animale et Biologie Intégrative, Domaine de Vilvert, 78350 Jouy-en-Josas, France; 2 CEA, DSV, IRCM, SREIT, Laboratoire de Radiobiologie et Etude du Génome, Domaine de Vilvert, 78350 Jouy-en-Josas, France; 3 AgroParisTech, Laboratoire de Génétique Animale et Biologie Intégrative, Domaine de Vilvert, 78350 Jouy-en-Josas, France; 4 Department of Animal Science, Center for Integrated Animal Genomics, Iowa State University, 2255 Kildee Hall, Ames, IA 50011-3150, USA; 5 INRA, UR892 de Virologie et Immunologie Moléculaires, 78350 Jouy-en-Josas, France; 6 INRA, UR66 de Pharmacologie-Toxicologie, 180 chemin de Tournefeuille, BP 93173, 31000 Toulouse, France

## Abstract

**Background:**

Designing sustainable animal production systems that better balance productivity and resistance to disease is a major concern. In order to address questions related to immunity and resistance to disease in pig, it is necessary to increase knowledge on its immune system and to produce efficient tools dedicated to this species.

**Results:**

A long-oligonucleotide-based chip referred to as SLA-RI/NRSP8-13K was produced by combining a generic set with a newly designed SLA-RI set that targets all annotated loci of the pig major histocompatibility complex (MHC) region (SLA complex) in both orientations as well as immunity genes outside the SLA complex.

The chip was used to study the immune response of pigs following stimulation of porcine peripheral blood mononuclear cells (PBMCs) with lipopolysaccharide (LPS) or a mixture of phorbol myristate acetate (PMA) and ionomycin for 24 hours. Transcriptome analysis revealed that ten times more genes were differentially expressed after PMA/ionomycin stimulation than after LPS stimulation. LPS stimulation induced a general inflammation response with over-expression of SAA1, pro-inflammatory chemokines IL8, CCL2, CXCL5, CXCL3, CXCL2 and CCL8 as well as genes related to oxidative processes (SOD2) and calcium pathways (S100A9 and S100A12). PMA/ionomycin stimulation induced a stronger up-regulation of T cell activation than of B cell activation with dominance toward a Th1 response, including IL2, CD69 and TNFRSF9 (tumor necrosis factor receptor superfamily, member 9) genes. In addition, a very intense repression of THBS1 (thrombospondin 1) was observed. Repression of MHC class I genes was observed after PMA/ionomycin stimulation despite an up-regulation of the gene cascade involved in peptide processing. Repression of MHC class II genes was observed after both stimulations. Our results provide preliminary data suggesting that antisense transcripts mapping to the SLA complex may have a role during immune response.

**Conclusion:**

The SLA-RI/NRSP8-13K chip was found to accurately decipher two distinct immune response activations of PBMCs indicating that it constitutes a valuable tool to further study immunity and resistance to disease in pig. The transcriptome analysis revealed specific and common features of the immune responses depending on the stimulation agent that increase knowledge on pig immunity.

## Background

Understanding resistance to disease is a major concern for all living organisms. Thus, it is necessary to design strategies to address related questions according to scientific and economic contexts. In farm animals like pig, zootechnical performances including growth, meat quality, feed intake or prolificacy have increased considerably during the last 25 years as a result of both the application of rational genetic selection schemes [1], and the improvement of feed formulations and sanitary conditions in breeding units. However at the same time, diseases have emerged that can cause substantial economic loss. Intensive research is carried out to better understand the etiology of emerging as well as endemic diseases in pig and raises questions on host pathogen interactions, pathogen latency, pathogen shedding, vaccine efficiency and host immune response. Thus, producing efficient methods and tools for these studies and improving basic knowledge on immune response in pig are major issues.

With the explosion of information on genome sequences and the emergence of functional genomics, it is now possible to study the expression of many genes in a single experiment. The development of DNA chips for genome-wide expression studies [2] and the next generation sequencing (NGS) technology for much deeper transcriptome analyses [3] are complementary approaches to conduct functional genomics research [4]. DNA chip-based transcriptome analyses are efficient to study host-pathogen interactions using either pathogen transcriptomes [5] or host transcriptomes [6-9] or both pathogen and host modifications of the transcriptome during infection [10]. Thus, DNA chips are still highly valuable to analyze large numbers of samples and in the case of domestic animals, it is essential to develop well-annotated DNA chips and sequence-based transcriptome using the NGS technology.

One major concern in designing a DNA chip-based experiment is to use the most appropriate and relevant array. For human and laboratory animals like mouse, the genomes are almost fully annotated, thus chips representing all the annotated genes are commercially available. In pig, the genome sequence is in progress and a first assembly has been released [11]. Today, many commercial and custom-made genome-wide microarrays exist for pig but probe annotation of these arrays is still poor because of the limited availability of full length cDNA sequences in pig [12]. Available porcine DNA chips include a 9 K cDNA-based microarray on nylon membranes [13], a 1789 DNA/cDNA microarray including a subset of probes specific for the SLA locus, a subset of immune response genes outside the SLA complex, and a subset of randomly chosen probes [10], the ARK-Genomics *Sus scrofa *Immune Array 3 K v1.0 [14], the *Sus scrofa *AROS (Array-Ready Oligo Sets) V1.1 (Operon Biotechnologies Inc., USA), the GeneChip^® ^Porcine Genome Array (Affymetrix, USA), a 25 K porcine long-oligonuclotide DNA microarray [15], and the Swine Protein-Annotated Oligonucleotide Microarray [16]. The immune system represents a complex network involving many regulation points and the genome-wide generic arrays that have been developed in pig only partially cover the genome and lack many immune response genes. As an example, the Major Histocompatibility Complex (MHC), which plays a key role in innate, adaptive immune response as well as in inflammation in mammals, is only poorly represented on existing pig expression arrays.

The objectives of our study were first to produce a generic array enriched in MHC and immunity-related genes and second to study transcriptome modifications of porcine peripheral blood mononuclear cells (PBMCs) after in vitro stimulation of the immune response. We describe the SLA-RI/NRSP8-13K chip that combines the generic Qiagen-NRSP8-13K set [17] with a long-oligonucleotide set comprising all the genes and pseudogenes annotated for the pig MHC referred to as the SLA (Swine Leucocyte Antigen) complex as well as immune response genes outside the SLA complex. We report the use of this array to investigate the differential expression of genes in PBMCs stimulated with lipopolysaccharide (LPS) or a mixture of Phorbol Myristate Acetate (PMA) and ionomycin for 24 hours. LPS is part of the outermost layer of gram-negative bacteria and is a pathogen-associated molecular pattern (PAMP) used for in vitro studies of the innate immune response after bacterial infection. PMA, a phorbol diester, is a potent tumor promoter often used in biomedical research to activate the signal transduction enzyme protein kinase C and a potent mitogen for PBMCs. Ionomycin is a ionophore that stimulates the intracellular production of the cytokines IL-2 and IL-4 in conjunction with PMA. Both these stimulations with either LPS or PMA/ionomycin were chosen because they are widely used to stimulate immune response in vitro. Our results show that some biological pathways and gene networks are differentially expressed in PBMCs according to stimulation. They provide new data on pig immunity and validate the relevance of the SLA-RI/NRSP8-13K chip for further studies on immunity and immune response to stimuli and pathogens in pig.

## Results

### Design of the porcine SLA-RI/NRSP8-13K chip

The porcine chip referred to as SLA-RI/NRSP8-13K chip includes a newly designed SLA-RI oligonucleotide set, the Qiagen-NRSP8 microarray oligonucleotide set [17] and a series of positive and negative control elements (Table 1). To prepare the SLA oligonucleotide subset, we selected the 151 genes and pseudogenes annotated in the SLA complex. This set comprises 816 probes: 410 probes in sense orientation and 406 probes in anti-sense orientation. The RI subset comprises 2957 probes of which 2832 are designed from pig sequences and 125 from human sequences. The detailed information on probes and on gene ontology (GO) is summarized in the Additional files 1 (SLA_RI_Table_S1.xls) and 2 (SLA_RI_Table_S2.doc), respectively. The SLA-RI set was uploaded into the animal QTL database [18] in order to visualize probes on existing porcine maps. Therefore, the position of probes from both SLA-RI and NRSP8-13K sets can be visualized via the QTLdb web viewer.

**Table 1:** Construction of SLA-RI/NRSP8-13K chip

Sets	Probes	Number of probes	Number of genes
**SLA-RI**			
	**SLA subset**		
	gene or pseudogenes	804	
	non-coding RNA	12	
	**RI subset**		
	Porcine sequence	2832	
	Human sequence	125	
	**Sub-total**	**3773**	**3104**

**NRSP8-13K**		**13297**	**8541**

**Control sets**	**Lucidea Universal ScoreCard**	92	
	**SpotReport Alien Control**	40	
	**Position or Negative Control**	1998	

**Total**		**19200**	**10010**^**1**^

^1 ^The total gene number is 10010 because 1635 genes are found in both gene sets

### Differentially expressed genes in PBMCs stimulated with LPS or PMA/ionomycin

**Figure 1:**
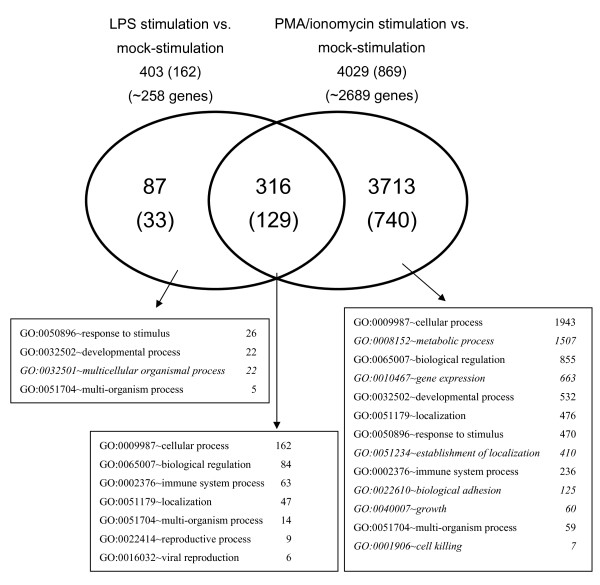
**Venn diagram representing the number of probes that were found differentially expressed after LPS or PMA/ionomycin stimulation compared to mock-stimulation**. The number of probes identified from the SLA-RI subset is indicated within brackets. The number of genes sharing the same GO is indicated in squares connected to the different parts of the diagram by arrows. GO annotations that were found specific to LPS or PMA/ionomycin stimulation are indicated in italic letters.

Transcriptome analyses were carried out using a dye-swap hybridization scheme to compare gene expressions between mock-stimulated PBMCs and PBMCs stimulated with either LPS or a mixture of PMA and ionomycin during 24 hours. Comparison of LPS stimulated and mock-stimulated PBMCs, revealed 403 differentially expressed probes (Figure 1 and Table 2) among which 162 originated from the SLA-RI set (40.2%) and 241 from the NRSP8-13K set (59.8%). Up-regulation was observed for 263 probes (65.3%) and down-regulation for 140 probes (34.7%). Comparison of PMA/ionomycin stimulated and mock-stimulated PBMCs, revealed 4029 differentially expressed probes (Figure 1 and Table 2) among which 869 originated from the SLA-RI set (21.6%) and 3160 from the NRSP8-13K set (78.4%). Up-regulation was observed for 2376 probes (59%) and down-regulation for 1653 probes (41%). Thus about ten times more genes are differentially expressed in PMA/ionomycin stimulated PBMCs than in LPS stimulated PBMCs. With both stimulation agents, more genes were up-regulated than down-regulated. We observed six GO annotations specific to PMA/ionomycin stimulation but only one specific to LPS stimulation (Figure 1). Comparison of the top-ten differentially expressed genes between the two stimulations applied (Table 3), found no common up-regulated gene, but five common down-regulated genes namely lysozyme (LYZ), fibronectin 1 (FN1), folate receptor 1 (FOLR1), cystatin C (CST3) and cystatin SA (CST2).

**Table 2:** Number of probes differentially expressed by PBMCs stimulated with LPS or PMA/ionomycin

Probe set	Regulation	LPS	PMA/ionomycin
SLA-RI	Up	98	377
	Down	64	492
	Sub-total	162	869
NRSP8-13K	Up	165	1999
	Down	76	1161
	Sub-total	241	3160

Total		403	4029

### Cluster analysis of common differentially expressed genes in PBMCs stimulated with LPS or PMA/ionomycin

**Figure 2:**
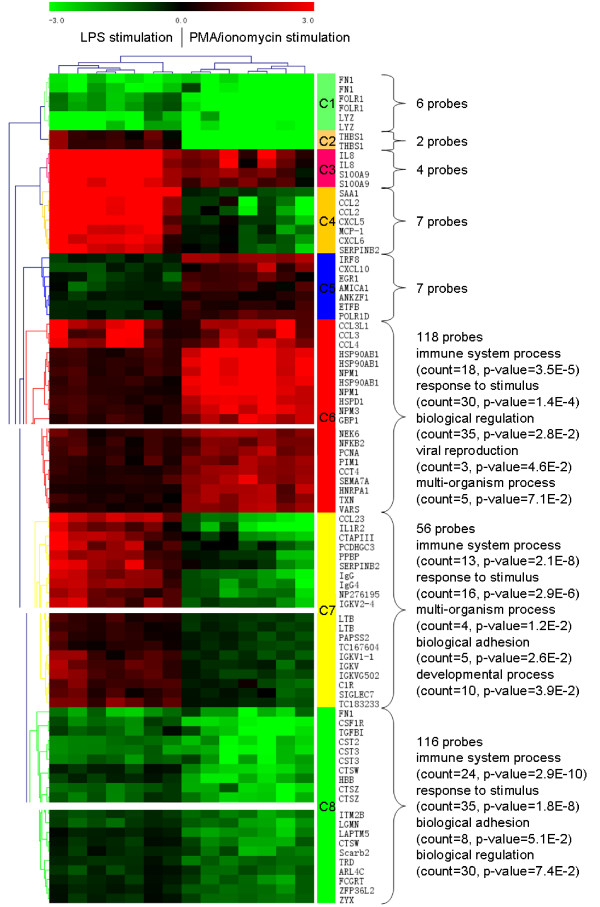
**Hierarchical clustering of the 316 probes found differentially expressed after LPS and PMA/ionomycin stimulations (Fisher p-value)**. The eight clusters C1 to C8 are partially represented. The whole clusters are provided in Additional file 4: SLA_RI_Figure_S4.png.

LPS and PMA/ionomycin stimulated PBMCs shared 316 differentially expressed probes (Figure 1) of which 244 were regulated in the same direction (124 up-regulated and 120 down-regulated), 65 up-regulated after LPS stimulation and down-regulated after PMA/ionomycin stimulation and seven down-regulated after LPS stimulation and up-regulated after PMA/ionomycin stimulation (Additional file 3: SLA_RI_Table_S3.doc). Hierarchical clustering classified these 316 probes into eight clusters C1 to C8 (Figure 2, and Additional file 4: SLA_RI_Figure_S4.png) and four cluster types. Clusters C1 and C8 contain probes down-regulated after either stimulation. Cluster C1 comprises six probes representing the three genes FN1, FOLR1 and LYZ, which are part of the top-ten most down-regulated genes (Table 3). Cluster C8 consists of 116 probes targeting at least 80 genes, which are involved in the following biological processes: immune system process, response to stimulus, biological adhesion and biological regulation. C8 includes MHC class II genes coding for light and heavy chains of the DR (DRA, DRB) and DQ series (DQA, DQB), the non classical MHC gene CD1, TGFB1, cystatin, cathepsin, and toll-like receptor TLR6 and TLR8 genes. Clusters C3 and C6 contain up-regulated genes after either stimulation. In cluster C3, up-regulation of genes was higher after LPS stimulation. This cluster contains four probes targeting the genes interleukin 8 (IL8 alias AMCF-I in pig) and S100 calcium binding protein A9 (S100A9) that belong to the top-ten most up-regulated genes after LPS stimulation (Table 3). Cluster C6 consists of 118 probes targeting at least 79 genes involved in the same biological processes as for cluster C8 with two more i.e. viral reproduction and multi-organism process. C6 includes many genes coding for heat shock proteins, CD44, chemokines, and proteasome-associated genes (Figure 2). The three clusters C2 (two probes for one gene), C4 (seven probes for five genes) and C7 (56 probes for 40 genes at least) group probes that were up-regulated after LPS stimulation and down-regulated after PMA/ionomycin stimulation whereas cluster C5 (seven probes for seven genes) contains probes down-regulated after LPS stimulation but up-regulated after PMA/ionomycin stimulation. The genes encoding immunoglobulins (IgM, IgA, IgG4, IgG2b, Igh-V) are found in cluster C7. Clusters C2 and C4 contain very few genes, mainly the most differentially expressed genes (Table 3), i.e. THBS1, SAA1, CCL2, CXCL5 and CXCL6.

**Table 3:** Top ten genes found differentially up- or down-regulated after LPS or PMA/ionomycin stimulation

	LPS stimulation			PMA/ionomycin stimulation		
	**Genes**	**Clusters**^**2**^	**Fold change**	**Genes**	**Clusters**^**2**^	**Fold change**

Up-regulation	SAA1	C4	26.84	IL2	No^3^	9.96
	IL8	C3	23.96	HSP90AB1	C6	8.49
	CCL2	C4	13.36	NPM1	C6	8.21
	S100A9	C3	13.33	CD69	No^3^	6.87
	CXCL5	C4	12.42	TNFRSF9	No^3^	6.62
	S100A12	No^3^	10.92	NPM3	C6	6.61
	CXCL3	No^3^	9.55	HSPD1	C6	6.51
	CXCL2	No^3^	7.96	GAPDH	C6	6.20
	CCL8	No^3^	7.36	RPS2	C6	5.31
	SOD2	C6	5.85	RAN	C6	5.31

Down-regulation	LYZ^1^	C1	-8.59	THBS1	C2	-65.98
	FN1^1^	C1	-7.57	FOLR1^1^	C1	-19.74
	SLA-DRB1	C8	-3.23	LYZ^1^	C1	-14.76
	FOLR1^1^	C1	-3.21	FN1^1^	C1	-12.26
	CST3^1^	C8	-2.80	CSF1R	C8	-8.15
	SLA-DQB1	C8	-2.74	TGFBI	C8	-6.78
	SLA-DOB	C8	-2.69	CST3^1^	C8	-6.51
	CST2^1^	C8	-2.67	CST2^1^	C8	-5.80
	SLA-DRA	C8	-2.65	CD163	No^3^	-5.59
	SLA-DQA1	C8	-2.40	CTSZ	C8	-5.58

^1 ^Genes that are commonly down-regulated after LPS or PMA/ionomycin stimulation

^2 ^The clusters refer to the hierarchical clustering reported in Figure 2 and Additional file 4

^3 ^"No" means that the genes were found differentially expressed in only one stimulation condition and were not included in the hierarchical clustering.

### Overview and comparison of affected biological functions in PBMCs during LPS or PMA/ionomycin stimulation

**Figure 3:**
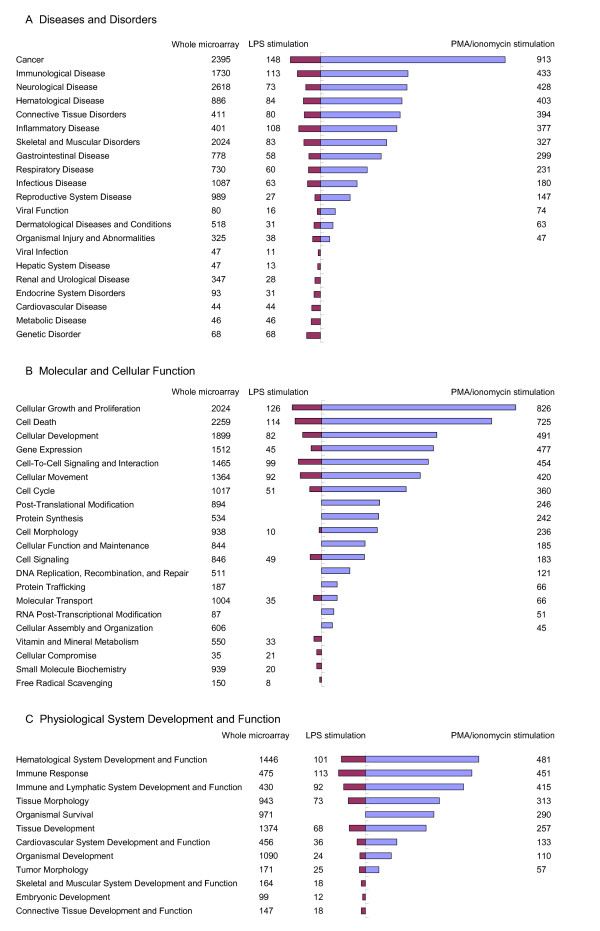
**Numbers of molecules in biological function categories related to the catalogs Diseases and Disorders (A), Molecular and Cellular Function (B) and Physiological System Development and Function (C) by IPA system**.

Three hundred and sixty-four genes from the 403 differentially expressed probes after LPS stimulation were mapped into the Ingenuity Pathway Analysis (IPA) system and 248 network eligible genes and 236 function eligible genes were found. Three thousand five hundred and sixty-eight genes from the 4029 differentially expressed probes found between mock-stimulated and PMA/ionomycin-stimulated PBMCs, were also mapped in the IPA system, leading to the identification of 2476 network eligible genes and 2115 function eligible genes. The numbers of molecules in each category of biological functions related to the different catalogs are given in Figure 3 and Table 4. In the catalog Diseases and Disorders, 21 and 14 biological function categories are covered respectively for LPS and PMA/ionomycin stimulations. The number of represented biological function categories after PMA/ionomycin stimulation is considerably reduced in comparison to LPS stimulation despite four times more differentially expressed genes. The two most represented biological function categories are common to both stimulations and concern first cancer and second immunological diseases (Figure 3A). In the catalog Molecular and Cellular Function, 14 and 17 biological function categories are covered respectively for LPS and PMA/ionomycin stimulations. The two most represented biological function categories are first cellular growth and proliferation and second cell death. In the case of LPS stimulation, some biological function categories are missing among which cellular assembly and organization, cellular function and maintenance and functions related to DNA replication, RNA modification and protein expression (Figure 3B). In the catalog Physiological System Development and Function (Figure 3C), 11 and 9 biological function categories are covered respectively for LPS and PMA/ionomycin stimulations. The three most represented functions are common to both stimulations and include immune system, hematological system development and function and immune and lymphatic system development and function. The function referred to as organismal survival is covered by a large set of 290 genes differentially expressed after PMA/ionomycin stimulation but is missing in the gene set differentially expressed after LPS stimulation. On the whole, after LPS stimulation, the relative representation of each catalog is 47% for Disease and Disorder, 30% for Molecular and Cellular Functions and 23% for Physiological System Development and Function while after PMA/ionomycin stimulation, the representation is 36% for Disease and Disorder, 43% for Molecular and Cellular Functions and 21% for Physiological System Development and Function. These results show that the predominant genes after LPS stimulation are those related to Disease and Disorder while after PMA/ionomycin stimulation, the most represented genes are related to Molecular and Cellular Functions.

**Table 4:** LPS and PMA/ionomycin stimulation: top biological functions from the catalogs Diseases and Disorders^1^, Molecular and Cellular Functions^2 ^and Physiological System Development and Function^3 ^identified by IPA and number of focus genes

Stimulation	Top biological functions	**# genes**^**4**^	Fisher p-value
LPS	Cellular Growth and Proliferation^2^	126/2404	2.25E-18 - 7.17E-07
	Cell Death^2^	114/2259	2.00E-27 - 9.59E-07
	Immunological Disease^1^	113/1730	3.40E-46 - 9.59E-07
	Immune Response^3^	113/475	6.00E-23 - 9.56E-07
	Inflammatory Disease^1^	108/401	2.58E-45 - 1.06E-06
	Hematological System Development and Function^3^	101/1446	6.70E-28 - 8.53E-07
	Cell-To-Cell Signaling and Interaction^2^	99/1465	2.44E-24 - 4.29E-07
	Cellular Movement^2^	92/1364	6.70E-28 - 8.33E-07
	Immune and Lymphatic System Development and Function^3^	92/430	3.43E-19 - 8.03E-07
	Skeletal and Muscular Disorders^1^	83/2024	2.52E-39 - -2.26E-07
	Cellular Development^2^	82/1899	1.66E-15 - 1.08E-06
	Connective Tissue Disorders^1^	80/145	2.52E-39 - -2.26E-07
	Tissue Morphology^3^	73/943	4.02E-20 - 8.53E-07
	Tissue Development^3^	68/1374	6.06E-18 - 5.30E-07
	Infectious Disease^1^	63/1087	1.60E-32 - 1.08E-06

PMA/ionomycin	Cancer^1^	913/2395	5.41E-25 - 5.07E-08
	Cellular Growth and Proliferation^2^	826/2404	3.15E-48 - 1.18E-08
	Cell Death^2^	725/2259	1.10E-32 - 5.07E-08
	Cellular Development^2^	491/1899	3.61E-32 - 4.90E-08
	Hematological System Development and Function^3^	481/1446	1.34E-35 - 4.73E-08
	Immune Response^3^	451/475	1.34E-35 - 4.73E-08
	Immunological Disease^1^	433/1730	1.83E-52 - 3.92E-08
	Immune and Lymphatic System Development and Function^3^	415/430	1.34E-35 - 2.91E-08
	Inflammatory Disease^1^	377/401	5.57E-36 - 4.26E-09
	Cell Cycle^2^	360/1017	4.35E-26 - 4.78E-08
	Skeletal and Muscular Disorders^1^	327/2024	5.57E-36 - 3.41E-08
	Tissue Morphology^3^	313/943	1.10E-32 - 2.30E-08
	Connective Tissue Disorders^1^	294/145	5.57E-36 - 5.29E-09
	Organismal Survival^3^	290/971	1.10E-15 - 2.41E-08
	Protein Synthesis^2^	242/534	1.30E-29 - 6.66E-09

^1 ^Diseases and Disorders

^2 ^Molecular and Cellular Functions

^3 ^Physiological System Development and Function

^4 ^Number of genes found differentially expressed after LPS or PMA/ionomycin stimulation divided by the total number of genes represented on the array for the biological functions

### LPS-related gene networks

**Figure 4:**
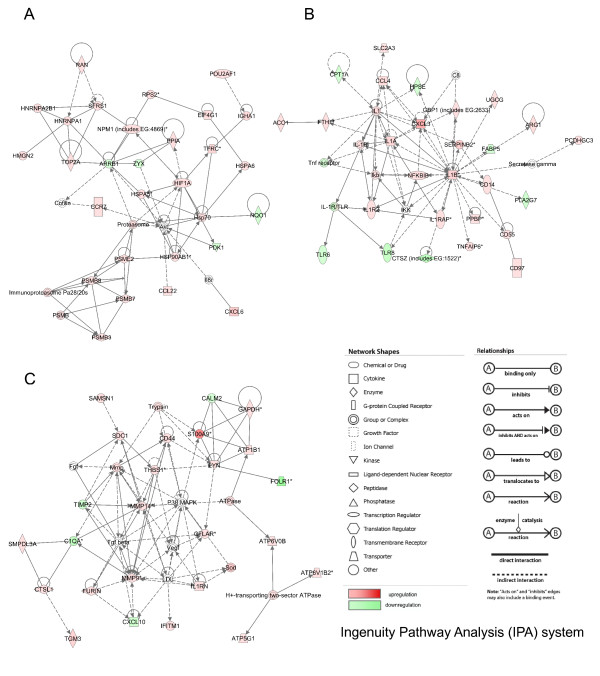
**LPS-related gene networks identified by IPA**. A: network 1, B: network 2, C: network 3. The network numbers refer to the numbers reported in Additional file 5: SLA_RI_Table_S5.xls.

Thirteen LPS-related gene networks with scores over 5 were built by the IPA system (Additional file 5: SLA_RI_Table_S5.xls). Significant gene networks specific to LPS stimulation are presented in Figure 4. The LPS-related network 1 was attributed the highest score (49) and groups 28 differentially expressed genes, among which 24 and four genes are up- and down-regulated, respectively (Figure 4A). This network is associated with various functions including cell death, immunological disease and molecular transport and covers canonical pathways, such as protein ubiquitination and antigen presentation pathways. This network represents an image of the up-regulation of proteasome activity and peptide processing in relationship to heat shock protein induction. The LPS-related network 2 groups 27 differentially expressed genes, among which 20 and seven genes are up- and down-regulated, respectively (Figure 4B). In this network, IL1B is in the central position together with IL1A and CXCL3 (chemokine (C-X-C motif) ligand 3). This network contains CD14 antigen (CD14), CD55 antigen (CD55), and CD97 antigen known to be a ligand of CD55 as well as TLR6 and TLR8. The LPS-related network 2 is an image of the global up-regulation of the CD14-induced cascade and cytokine signaling pathways related to steps specific to innate immune response after LPS stimulation in association to a down-regulation of TLR6 and TLR8. The LPS-related network 3 consists of 25 differentially expressed genes, among which 20 and five are up- and down-regulated, respectively (Figure 4C). The network is associated with functions linked to immunological disease, cancer, renal and urological diseases, and concerns a series of six canonical pathways, including purine metabolism, oxidative phosphorylation, glucocorticoid receptor signaling, IL-8 signaling, hepatic fibrosis/hepatic stellate activation and leukocyte extravasation signaling. The MAP kinase MAP14 (alias P38MAPK), the matrix metallopeptidases 9 and 14 (MMP9 and MMP14), the transforming growth factor beta 1 (TGFB1) are in central positions in this network, which groups up-regulated genes involved in intracellular biochemistry modifications and in remodeling.

**Figure 5:**
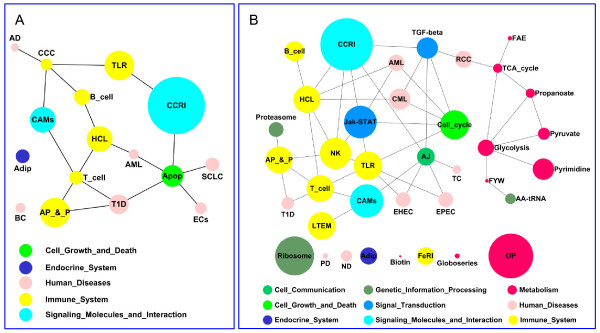
**Interactions of KEGG biological pathways identified with genes found differentially expressed after LPS (A) or PMA/ionomycin stimulation (B)**. The pathway symbols are indicated in Table 5. The circle size allocated to the pathways is proportional to the number of genes in each pathway.

Complementary information on gene pathways and on interactions between pathways was retrieved using the Kyoto Encyclopedia of Genes and Genomes (KEGG) [19]. After LPS stimulation, 16 significant pathways with a Fisher Exact P-Value < 0.05 were identified (Table 5, and Additional file 6: SLA_RI_Table_S6.doc). The cytokine-cytokine receptor interaction pathway is the most representative pathway followed by antigen processing and presentation and Toll-like receptor signaling pathways. Interactions between pathways with their relative importance are presented in Figure 5A. Fourteen pathways are interconnected and only adipocyte signaling and bladder cancer pathways are not connected to the other pathways.

**Table 5:** List of KEGG biological pathways associated with the genes differentially expressed after LPS or PMA/ionomycin stimulation

Stimulation	KEGG pathway	Symbol	**# genes**^**1**^	Fisher p value
LPS	Cytokine-cytokine receptor interaction	CCRI	29/233	1.00E-08
	Antigen processing and presentation	AP_&_P	15/57	3.10E-07
	Toll-like receptor signaling pathway	TLR	15/89	1.88E-06
	Cell adhesion molecules	CAMs	13/120	6.85E-04
	Hematopoietic cell lineage	HCL	13/83	9.03E-06
	Apoptosis	Apop	11/76	1.41E-04
	Type I diabetes mellitus	T1D	10/31	1.88E-06
	B cell receptor signaling pathway	B_cell	8/67	1.60E-03
	Adipocytokine signaling pathway	Adip	7/58	1.25E-02
	Small cell lung cancer	SCLC	7/126	3.21E-02
	T cell receptor signaling pathway	T_cell	7/99	4.37E-02
	Complement and coagulation cascades	CCC	6/69	3.30E-02
	Epithelial cell signaling in Helicobacter pylori infection	ECs	6/59	3.10E-02
	Acute myeloid leukemia	AML	5/53	4.84E-02
	Bladder cancer	BC	5/39	1.49E-02
	Alzheimer's disease	AD	4/122	1.55E-02

PMA/ionomycin	Cytokine-cytokine receptor interaction	CCRI	74/233	4.86E-02
	Oxidative phosphorylation	OP	63/96	0.00E+00
	Ribosome	Ribosome	55/78	0.00E+00
	Cell adhesion molecules	CAMs	47/120	1.98E-03
	Jak-STAT signaling pathway	Jak-STAT	46/129	4.96E-02
	Natural killer cell mediated cytotoxicity	NK	44/102	9.13E-03
	Cell cycle	Cell_cycle	41/93	3.11E-03
	Toll-like receptor signaling pathway	TLR	40/89	4.14E-04
	Leukocyte transendothelial migration	LTEM	39/108	1.36E-02
	Hematopoietic cell lineage	HCL	38/83	5.22E-05
	T cell receptor signaling pathway	T_cell	38/99	2.16E-04
	Antigen processing and presentation	AP_&_P	37/57	7.05E-05
	Chronic myeloid leukemia	CML	30/64	1.88E-03
	Pyrimidine metabolism	Pyrimidine	30/68	2.07E-02
	TGF-beta signaling pathway	TGF-beta	30/69	2.82E-02
	B cell receptor signaling pathway	B_cell	28/67	3.85E-04
	Adherens junction	AJ	25/68	4.27E-02
	Fc epsilon RI signaling pathway	FeRI	25/66	4.27E-02
	Adipocytokine signaling pathway	Adip	24/584	4.65E-02
	Renal cell carcinoma	RCC	24/62	2.84E-02
	Acute myeloid leukemia	AML	23/53	4.47E-03
	Glycolysis/Gluconeogenesis	Glycolysis	23/40	1.76E-02
	Pathogenic Escherichia coli infection - EHEC	EHEC	21/42	8.24E-03
	Pathogenic Escherichia coli infection - EPEC	EPEC	21/42	8.24E-03
	Proteasome	Proteasome	21/43	0.00E+00
	Type I diabetes mellitus	T1D	18/31	9.62E-03
	Pyruvate metabolism	Pyruvate	17/31	1.32E-02
	Neurodegenerative Disorders	ND	16/32	1.38E-02
	Aminoacyl-tRNA biosynthesis	AA-tRNA	15/28	2.47E-02
	Citrate cycle (TCA cycle)	TCA_cycle	13/27	1.12E-02
	Propanoate metabolism	Propanoate	13/23	4.57E-02
	Thyroid cancer	TC	12/26	2.91E-02
	Prion disease	PD	8/34	7.98E-03
	Glycosphingolipid biosynthesis - globoseries	Globoseries	7/43	3.12E-02
	Fatty acid elongation in mitochondria	FAE	6/10	1.62E-02
	Phenylalanine, tyrosine and tryptophan biosynthesis	FYW	5/10	4.18E-02
	Biotin metabolism	Biotin	3/7	4.56E-02

^1 ^Number of genes found differentially expressed after LPS or PMA/ionomycin stimulation divided by the total number of genes represented on the array for the KEGG pathways

### PMA/ionomycin-related gene networks

**Figure 6:**
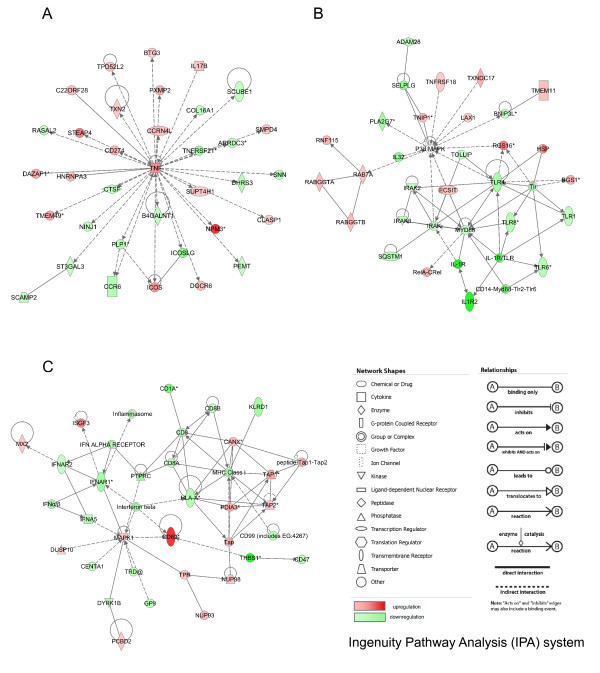
**PMA/ionomycin-related gene networks identified by IPA**. A: network 1, B: network 53, C: network 57 in which THBS1 and CD47 were assembled by connection between CD69 and THBS1 in network 94 that was not presented here. The network numbers refer to the numbers reported in Additional file 7: SLA_RI_Table_S7.xls.

Ninety-eight PMA/ionomycin-related biological networks with a score higher than 5 were built by the IPA system (Additional file 7: SLA_RI_Table_S7.xls). A limited number of the most interesting networks is shown on Figure 6. In the PMA/ionomycin-related network 1 (Figure 6A), 19 and 16 genes are up- and down-regulated, respectively. Tumor necrosis factor (TNF) is significantly over-expressed after PMA/ionomycin stimulation and occupies the most central position in the network. The network is linked with numerous functions including cellular development, cellular growth and proliferation, hematological system development and function and concerns about 30 canonical pathways representative of the pleiotropic activities of TNF. Since most pathways were identified by a single gene (mostly TNF), it was not possible to connect this TNF-centered network with a specific biological function or pathway modified by PMA/ionomycin stimulation. Thus, we searched for networks with canonical pathways related to more than one or two genes. Network 53 (Figure 6B) groups 20 down-regulated genes, including TLR genes (TLR1, TLR6, TLR8, TLR4), myeloid differentiation primary response gene 88 (MYD88), interleukin-1 (IL1), receptor-associated kinases (IRAK, IRAK1 and IRAK3) and IL1 receptors (IL1R and IL1R2). Network 53 also contains 14 up-regulated genes that comprise members of the TNF-receptor superfamily (TNFRSF18) and Ras-associated proteins such as RAB7. The non-differentially expressed P38MAPK gene occupies the central position and connects the down-regulation of the TLR pathway to the up-regulation of TNF receptors and vesicular transport. Network 57 (Figure 6C) comprises 20 down-regulated genes mostly associated to MHC class I presentation and interferon signaling pathways, and 15 up-regulated genes including genes involved in peptide processing before loading onto class I molecules (ABC transporters TAP1 and 2, calnexin CANX). The most up-regulated gene i.e. CD69 is found in this network and could be connected to the most down-regulated gene THBS1 from network 94 and to CD47. Therefore, the network presented in Figure 6C clearly connects the strong up-regulation of a cell surface glycoprotein involved in lymphocyte proliferation and known to act as a signal-transmitting receptor in lymphocytes, natural killer (NK) cells, and platelets [20] and the very strong down-regulation of THBS1 that is an adhesive glycoprotein involved in cell-to-cell and cell-to-matrix interactions. Moreover, this network provides evidence for the co-existence of an up-regulation of genes involved in peptide processing and a down-regulation of genes involved in antigen presentation to the cell surface.

After PMA/ionomycin stimulation, 37 KEGG pathways with a Fisher Exact P-Value < 0.05 were identified (Table 5, and Additional file 6: SLA_RI_Table_S6.doc). The most represented pathways are cytokine-cytokine receptor interaction, oxidative phosphorylation, ribosome, cell adhesion molecules (CAMs), Jak-STAT signaling pathway, natural killer cell mediated cytotoxicity and cell cycle. The Toll-like receptor signaling pathway occupies the eighth position with 40 genes. Interactions between pathways with their relative importance are presented in Figure 5B. Globally, PMA-ionomycin stimulation mostly modifies pathways associated with the immune system, signaling molecules and interactions, human diseases and metabolism like LPS stimulation but it also affects additional pathways associated to metabolism, cell growth and death and signal transduction.

### Expression of probes mapping to the SLA complex

Since sense and antisense probes for all SLA annotated transcripts were present on the DNA chip (Table 1), it was possible to perform an in-depth analysis of the expression profile of all annotated transcripts mapping to the locus. For probes targeting protein coding genes, only the differential expression was studied. For antisense and non-coding transcripts, expression and differential expression between stimulation and mock-stimulation were both analyzed.

All the differentially expressed genes belonging to the MHC antigenic processing and presentation pathway are listed in Table 6. After LPS stimulation, all the genes associated with the MHC class II mediated peptide presentation pathway are down-regulated. The down-regulated genes include genes involved in peptide processing in the late endosome (CTSB, CTSS, LGMN), peptide loading (CD74 alias CLIP) and peptide presentation to the CD4 T cells (SLA class II genes DRA, DRBs, DQA, DQBs). After PMA/ionomycin stimulation, a similar down-regulation of MHC class II mediated peptide presentation pathway is observed. In contrast, for the MHC class I mediated peptide presentation pathway, all genes involved in peptide processing and transport (PSMB5 alias LMPX, PSMB6 alias LMPY, PSMB8 alias LMP7, PSMB9 alias LMP2, TAP1, TAP2, CALR and CANX) are up-regulated whereas probes targeting the classical class I genes SLA-3, the non classical class I genes SLA-6 and SLA-7 as well as pseudogenes SLA-4 and SLA-11 are down-regulated.

**Table 6:** List of differentially expressed genes in MHC class I and class II presentation pathways after stimulation by LPS or PMA/ionomycin

Stimulation	Pathway	Gene symbol (Pig)	Fold Change
LPS	MHC I pathway	HSP90AB1	1.44
		HSPA5	1.41
		PSMB8	1.27
		PSME2	1.33
	
	MHC II pathway	CD74	-2.01
		CTSB	-1.38
		CTSL1	2.80
		CTSS	-1.61
		LGMN	-1.81
		SLA-DMA	-1.82
		SLA-DMB	-2.12
		SLA-DOB	-2.69
		SLA-DQA	-2.40
		SLA-DQB	-2.34
		SLA-DRA	-2.50
		SLA-DRB1	-2.28
		SLA-DRB3	-1.68

PMA/ionomycin	MHC I pathway	CALR	1.84
		CANX	1.64
		HSP90AA1	2.06
		HSP90AB1	7.72
		HSP90B1	1.87
		HSPA1A	1.52
		HSPA1B	-1.27
		HSPA1L	-1.33
		HSPA4	1.55
		HSPA5	1.84
		HSPA6	3.46
		HSPA8	4.16
		HSPA9	2.95
		HSPBP1	1.66
		IFNA5	-1.48
		LTA	2.18
		PDIA3	1.79
		PSMB5	2.68
		PSMB6	2.55
		PSMB8	2.10
		PSMB9	2.43
		PSME1	2.16
		PSME2	2.56
		SLA-3	-1.29
		SLA-4	-1.26
		SLA-6	-2.01
		SLA-7	-1.29
		SLA-11	-1.95
		TAP1	1.85
		TAP2	1.41
		TNF-a	2.83
		TRAP1	1.53
	
	MHC II pathway	CD74	-2.40
		CTSB	-2.24
		CTSS	-3.01
		LGMN	-3.71
		SLA-DMA	-2.06
		SLA-DMB	-2.31
		SLA-DOA	-1.38
		SLA-DOB	-2.50
		SLA-DQA	-3.14
		SLA-DQB	-2.62
		SLA-DRA	-2.83
		SLA-DRB1	-3.12
		SLA-DRB3	-2.05
		SLA-DRB5	-1.35

In order to analyze anti-sense oligonucleotide and non-coding RNA probe expression, the A value (A = 1/2(log_2_(Cy3*Cy5))) was used. Since the average A value of probes corresponding to negative controls was 7.8, probes were considered as expressed for A values higher than 8.8 that corresponded to signal intensities twice as high as for the controls. With such a threshold, about 30% of the anti-sense oligonucleotide probes were found expressed. After LPS stimulation, 135 probes corresponding to anti-sense sequences derived from 93 genes are expressed. After PMA/ionomycin stimulation, 124 probes corresponding to anti-sense sequences from 85 genes are expressed among which 121 are expressed by PBMCs in both stimulation conditions. Anti-sense sequences of eight genes (ABCF1, C7H6orf27, LTA, NRM, SFTPG, snoRNAU52 (RF00276), SLA-1 and SLA-DOB) are specifically expressed in LPS-stimulated PBMCs. For non-coding RNA, sense probes targeting mir-219 (RF00251) and snoRNAU84 are expressed by PBMCs stimulated by LPS or PMA/ionomycin and the anti-sense probe targeting snoRNAU52 (RF00276) is specifically expressed in LPS-stimulated PBMCs. Differential analysis revealed that no non-coding RNA is differentially expressed whatever the stimulation and that antisense probes are regulated only after PMA/ionomycin stimulation. Four probes are up-regulated (anti-sense sequences of BAT3, IER3, EGFL8 and PSMB9) and nine probes are down-regulated (anti-sense sequences of OLF42-3, STK19, LSM2, AIF1, STK19, BAT1, RPP21, SKIV2L and PPT2).

### Validation of differentially expressed genes at the RNA level

**Figure 7:**
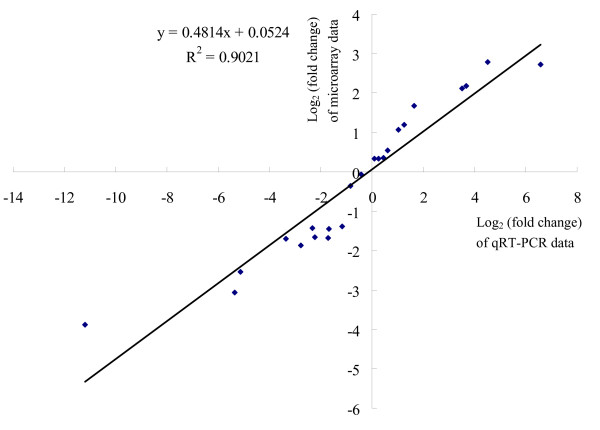
**Comparison between microarray-based and qRT-PCR-based results by Pearson correlation scattered plots**.

Differential expression of 14 genes (Table 7) was validated by quantitative real-time PCR (qRT-PCR) and the B2M gene was included as a reference gene for data normalization. In order to strengthen the comparison between both technologies, qRT-PCRs were carried out using the RNA samples that were used for microarray experiments and the fold change was calculated for both microarray and qRT-PCR data (Additional file 8: SLA_RI_Table_S8.doc). For MHC mediated peptide presentation, five genes involved in the peptide processing and presentation by MHC class I molecules (PSMB8, PSMB9, SLAIa, TAP1 and TAP2) and three genes involved in the processing and presentation of antigens by MHC class II molecules (SLA-DQB, SLA-DRA and SLA-DRB1) were chosen. Three genes CST2, LYZ and PPIA were chosen for validation because they were differentially expressed in opposite directions after LPS or PMA/ionomycin stimulation. IL1A was chosen because it was differentially expressed only after LPS stimulation and inversely, CD69 and TNFRSF9 were chosen because they were differentially expressed only after PMA/ionomycin stimulation. Differential expression was confirmed for all genes and the log_2_(fold change) calculated with the qRT-PCR data consistently showed a greater magnitude of change compared to the log_2_(fold change) calculated with the microarray data. A highly significant correlation (R^2 ^= 0.9021, p value < 10E-6) was calculated between the two techniques (Figure 7).

**Table 7:** Primer sequences for qRT-PCR validation

Gene name (Symbol)	Forward	Reverse	Amplicon size (bp)	Accession number
B2M^1^	TGGTCTTTCTACCTTCTGGTCC	TGTGATGCCGGTTAGTGGTCTC	134	NM_004048
CD69	TCCTATCCCATGTGCTGTGGT	GCACATATTGGCCTGGACAGT	104	NM_214091
CST2	TCTTGGAAGTGGAGATTGGCC	CACAGCGTTTTCTTCTGCAGG	103	BG609449
IL1A	GTGTGGTGATGGCAGCAGC	GGTCGTCATCGGTGATGAACT	81	NM_214029
PSMB9	GCGCTTCACCACAAATGCTA	TCCACACCAGCAGCTGTAATG	96	NM_001037961
PSMB8	TACCTGCTTGGCACCATGTCT	AGTACAACCTGCACTCCTTGGC	82	NM_213935
LYZ	TCTTGCGCTTCTCCTCCTTTC	AAACATACCCAGTTCGCCAGG	129	NM_214392
PPIA	GTCTCCTTCGAGCTGTTTGCA	CCAAATCCTTTCTCCCCAGTG	83	NM_214353
SLAIa	CATCATTGTTGGCCTGGTTC	CCTTTTTCACCTGAGCGC	90	NM_001097431(SLA-1) NM_001113702(SLA-2) NM_001097427(SLA-3)
SLA-DQB	AGGAGATGTCTACACCTGCCG	ATTCAGACTGTGCCCGCC	80	NM_001113694
SLA-DRA	ATGGGCTATCGTAGAGAATCACG	CAAACATAAACTCGCCAGATTTG	80	NM_001113706
SLA-DRB1	CACAGTGGAATGGAGGGCAC	AGCAGACCCAGGACGAAGC	81	NM_001113695
TAP1	CCAGTATCTCAGGGATGTTGCTG	CGCTGCTTATAGCCCCACC	81	NM_001044581
TAP2	TGTTGGGTGAGACACTAATCCCTTA	CAAAGGCATCAGGGTCAAAATC	81	DQ227991
TNFRSF9	TCCAGGTCACACTTCCCATGT	GAACAACAGAGAAACGGAGCG	101	BE233113

^1 ^Reference gene for qRT-PCR

### Validation of differentially expressed genes at the protein level

Supernatants of mock-stimulated PBMCs and PBMCs stimulated with LPS or PMA/ionomycin for 24 hours were collected to measure cytokines IL-8, IL-12, TNFA and IL-1B by enzyme-linked immunosorbent assay (ELISA) tests. Gene expression between mock-stimulation and each stimulation condition assessed by the fold change was calculated for both microarray and ELISA data (Table 8). Significant increased expression of IL8, IL12, TNFA and IL1B proteins (p < 0.05) were detected after both stimulations and confirmed up-regulation for IL8 and IL1B at the RNA level after LPS stimulation and up-regulation of IL8, IL12 and TNFA at the RNA level after PMA/ionomycin stimulation. High discrepancies were observed between RNA and protein levels for IL8, IL12, TNF and IL1B. For LPS stimulation, RNA levels of IL12 and TNFA were not significantly different between mock-stimulated and stimulated cells in contrast to the protein levels. Similarly, for PMA/ionomycin stimulation, RNA level of IL1B was not significantly up-regulated in contrast to the protein level. Such discrepancies could be due to underestimation of RNA levels due to the sensitivity scale of the transcriptome analysis or to specific properties of the proteins including lability, half life time, differences in release timing, and accumulation of the released proteins in the supernatants.

**Table 8:** Comparison of gene expression fold change between stimulated and non stimulated PBMCs at the protein (ELISA tests) and RNA levels (microarray)

	LPS stimulation fold change^1^		PMA/ionomycin stimulation fold change^1^	
	**Microarray**	**ELISA**	**Microarray**	**ELISA**

IL8	23.95	105.79	3.12	36.73
IL12	1.03^NS^	7.66	1.43	7691.24
TNFA	1.23^NS^	7.48	2.83	1126.79
IL1B	5.00	144.81	1.04^NS^	136.62

^1^NS: not significant at p value < 0.05.

Differential expression of MHC class I and class II molecules was validated by fluorescence-activated cell sorting (FACS). FACS analysis confirmed a significant down-regulation of MHC class I molecules at the surface of PBMCs stimulated with PMA/ionomycin for 24 hours compared to mock-stimulated PBMCs. The MHC class I mean fluorescence intensity of PBMCs after PMA/ionomycin stimulation was 52.6% of that of mock-stimulated PBMCs (p = 0.0096). As expected from microarray results, no change in MHC class I molecule expression was detected at the surface of LPS stimulated PBMCs for 24 hours. In contrast, MHC class II molecules were found down-regulated at the surface of PBMCs in both stimulation conditions compared to mock-stimulated cells. The MHC class II mean fluorescence intensity of LPS-stimulated PBMCs was 68.9% of that of mock-stimulated PBMCs (p = 0.033) and 72.1% of that of mock-stimulated PBMCs (p = 0.054) after PMA/ionomycin stimulation.

## Discussion

The objectives of this study were first to produce a well annotated and an easy to use DNA chip to analyze the immune response in pig and second to validate its relevance by investigating transcriptome modifications in PBMCs stimulated with LPS or PMA/ionomycin for 24 hours. The same seven biological replicates from seven distinct animals were used for transcriptome analysis, qRT-PCR and ELISA validation, and another set of seven animals was used for validation by FACS analysis. Reproducibility of the results was good.

### Relevance of the SLA-RI/NRSP8-13K chip

DNA chips targeting immunity have been reported for human, mouse and a few domestic species including cow [21,22], chicken [23,24] and to a lesser extent pig with a unique report of a nylon membrane comprising less than 100 genes [25]. Designing dedicated chips may be criticized because it is contradictory to the global approach that underlies a transcriptome study. Since no genome-wide expression array exists for pig and since efficient tools are required to study immunity and resistance to disease, we have constructed a generic array enriched in immunity genes. We combined a well-annotated oligonucleotide set referred to as the NRSP8-13K set that partially covers the pig genome [17] to a set of oligonucleotides referred to as the SLA-RI set that targets all annotated loci within the SLA complex and immunity genes outside the SLA complex. Here, we report that after LPS stimulation, among 258 differentially expressed genes (403 probes, see Table 2), 61 were common to both the generic and the SLA-RI sets and 84 were present only in the SLA-RI set. Similarly, after PMA/ionomycin stimulation, among 2689 differentially expressed genes, 353 were present in both sets and 424 were present only in the SLA-RI set. The SLA-RI set was highly informative for the analyses reported here. The SLA-RI set may be merged with any other generic set and it is anticipated that the number of overlapping probes between sets should increase as a function of the genome coverage in the next generation expression arrays. The SLA-RI/NRSP8-13K chip was shown to be suitable to identify immunity and disease-related biological pathways and functions as well as to construct relevant gene networks. Validation of differential expression was carried out for several genes at the RNA level by qRT-PCR and at the protein level by ELISA tests or FACS analysis. The results show significant correlations between mRNA and protein expression levels, confirming the accuracy of the chip annotation. DNA chips for expression studies are currently replaced by sequence-based transcriptome using the NGS technology, suggesting that the design of genome-wide DNA chips could be skipped and that sequencing could be used directly for transcriptome analysis. However, concentrating all efforts on the NGS technology might hamper the analysis of numerous animals and samples as required for eQTL studies and genetic genomics [26]. We are quite convinced that the NGS technology and well-annotated DNA chips will remain complementary for a while in domestic species. The SLA-RI/NRSP8-13K chip reported here represents an accurately annotated chip dedicated to the pig immune system and will provide a valuable tool for diagnostics and research.

### Choice of the in vitro models to study immune response activation

PMA, also known as 12-O-tetradecanoylphorbol-13-acetate (TPA), is a potent tumor promoter often used in biomedical research [27]. Ionomycin is an ionophore produced by *Streptomyces conglobatus*. PMA in conjunction with ionomycin is known to activate T and B cells and has been used in numerous immune-related studies [25,28,29]. LPS is a major structural component of the outer membrane of gram-negative bacteria and is a well-referenced PAMP. LPS stimulation of mammalian cells occurs through a series of interactions with proteins including LPS binding protein, CD14, MD-2 and TLR4 [30]. LPS is one of the best studied immunostimulatory components of bacteria and can induce systemic inflammation and sepsis if excessive signals occur [31]. LPS-stimulation mimics a bacterial infection and has been extensively used to study innate immune response [31,32]. Two recent studies in pig have reported transcriptome modifications in mesenteric lymph node or spleen after infection by *Salmonella enterica *serovar Choleraesuis (*S. Choleraesuis*) [8] and *Haemophilus parasuis *(*H. parasuis*) [9], respectively. *S. Choleraesuis *and *H. parasuis *are both gram-negative bacteria. Our results on LPS-stimulation reveal that many genes already identified after in vivo infection by *S. Choleraesuis *and *H. parasuis *are up- or down-regulated confirming that in vitro LPS activation of PBMCs is a good model to study innate immune response to infection with gram negative bacteria in pig.

Indeed, LPS and PMA/ionomycin stimulations were chosen because they are widely used as gold standard in vitro models to measure cytokines released in the medium by PBMCs in many species. A unique time point was studied and we are aware that all the results reported here correspond to this unique time point i.e. 24 hours after stimulation. It has been reported that time points earlier than 24 hours are more relevant to decipher the onset of the response to stimulus as shown in kinetics studies in cow [22], pig [25], mouse [32] or human [33]. Moreover, kinetics studies have revealed that many genes return to their basal expression level by 48 hours of stimulation, suggesting that homeostasis is restored at that time [22,25]. In this report, we were interested in studying the PBMC transcriptome at the time when cytokines released in the medium are efficiently measured. Our results provide many candidate genes to test for kinetics studies and ongoing complementary studies focus on this topic.

Significant positive correlations have been reported between transcriptomes of total PBMCs and purified monocytes stimulated with LPS, suggesting that for studies focussing on the most differentially expressed genes, separating and analysing cell subpopulations may be unnecessary [34]. Therefore, the results reported here correspond to the most striking transcriptome modifications during immune response activation and may miss some subtle changes that occur in each cell subtype. Identifying transcriptome modifications occurring in each cell subtype is a major objective to better decipher immune response. However, transcriptomic signatures of blood or total PBMCs are of high interest in clinical research and most studies relate to total PBMCs in pig [35].

### Specific transcriptome modifications after LPS stimulation

Almost half of the transcriptome modifications due to LPS stimulation are related to Disease and Disorder biological function (see Figure 3). Most of the up-regulated genes relate to inflammation and innate immune response, as expected. SAA1 and pro-inflammatory chemokines IL8, CCL2, CXCL5, CXCL3, CXCL2 and CCL8 belong to the top-ten most up-regulated genes (see Table 3), SAA1 being the most up-regulated gene with a 27-fold change by comparison to mock-stimulated PBMCs. SAA1 encodes the major acute-phase protein Serum Amyloid A (SAA), the precise role of which is still unclear despite reports suggesting a key role in the establishment and maintenance of inflammation notably as an antiapoptotic agent for neutrophils [36] and as an opsonin that would facilitate phagocytosis of gram-negative bacteria [37]. SAA1 was also found as the most up-regulated gene in spleen seven days after infection by *H. parasuis *[9]. The chemokines IL8, CXCL5, CXCL3 and CXCL2 have chemotaxis for neutrophils whereas the chemokines CCL2 and CCL8 have a broader chemotaxis spectrum specific for T, dendritic and NK cells as well as monocytes and basophils [38]. Up-regulation of IL8 has already been reported in pig PBMCs [39,40] and amnion [41] after bacterial infection. In human, stimulation of PBMCs with LPS induces the secretion of CCL2 [42], CXCL3 and CXCL2 [43]. CXCL5 is up-regulated in LPS-challenged bovine mammary epithelial cells [44]. All these results confirm the essential role of chemokines in chemoattraction and cell guidance to the site of infection during bacterial infection. IL1 has been reported to activate chemokine production [45]. In our study, we found that IL1 was moderately up-regulated after 24 hours of stimulation and that it occupies a central position in the LPS-related network 2 providing a global image of inflammation activation (Figure 4B).

We have also found other strongly up-regulated genes after LPS stimulation including SOD2, S100A9 and S100A12. S100A9 and S100A12 are members of the S100 family, which encodes proteins containing two EF-hand calcium-binding motifs and are involved in the regulation of a number of cellular processes such as cell cycle progression and differentiation. In human, S100A9 has been reported to be up-regulated in LPS-stimulated bronchial epithelial cells [46], suggesting that this gene has a role in innate immune defense. SOD2 (superoxide dismutase 2) is a member of the iron/manganese superoxide dismutase family and it has been shown to be up-regulated in dendritic cells after LPS stimulation [47]. These results suggest that the calcium pathways as well as oxidative processes are strongly affected in LPS stimulated PBMCs. Interestingly, the six genes S100A9, CXCL5, S100A12, IL8 CXCL2 and SOD2 were also found strongly up-regulated in mesenteric lymph nodes of pigs infected by *S. Choleraesuis *[8] and S100A9, S100A12 and SOD2 were also up-regulated in spleen after *H. parasuis *infection [9]. Overall these results confirm a predominant role of common genes in the innate immune response of pig to gram-negative bacterial infections.

### Specific transcriptome modifications after PMA/ionomycin stimulation

By examining the top-ten most up-regulated genes (Table 3) and the representation of KEGG pathways (see Table 5 and Figure 5B), we have found that after PMA/ionomycin stimulation, T cells are more activated than B cells. Three of the most up-regulated genes are IL2 (fold change close to 10), CD69 (fold change = 6.9) and TNFRSF9 (fold change = 6.6), which are related to T cell activation. B cell markers such as CD40 and INHBA are also up-regulated (Additional file 9: SLA_RI_Table_S9.xls) but with fold changes of 1.6 and 5, respectively. It has been reported that transcription of the cytokine IL2 is the main consequence of T cell activation and that IL2 is produced by T helper cells harboring a Th1 cytokine profile [38]. IL2 is essential for the generation and regulation of immune response. Binding of IL2 activates the Ras/MAPK, JAK/Stat and PI 3-kinase/Akt signaling module pathways (IPA, Ingenuity). IL2 signals through the IL2 receptor and in this study, we have found that the interleukin 2 receptor alpha (IL2RA) is also up-regulated after PMA/ionomycin stimulation as previously reported [25]. The IL-2/IL-2R interaction stimulates the growth, differentiation and survival of antigen-selected cytotoxic T cells [48]. The activation of T lymphocytes, both in vivo and in vitro, induces expression of CD69 that has been reported as the earliest inducible cell surface glycoprotein acquired during lymphoid activation. This molecule is involved in lymphocyte proliferation and functions as a signal-transmitting receptor in lymphocytes, natural killer cells and platelets [20]. TNFRSF9 also known as 4-1BB is a member of the TNF-receptor superfamily and is a CD4+ T cell marker that regulates CD28 co-stimulation to promote Th1 cell response. The expression of this receptor is induced by lymphocyte activation and is involved in T cell division and expansion [49]. In agreement with these findings, IL12B that is known to trigger Th1 response is also found up-regulated with a limited fold change of 1.5. Globally, our results show a strong up-regulation of cytokines and genes related to Th1 response, suggesting a more pronounced activation of the Th1 response compared to a Th2 response after PMA/ionomycin stimulation for 24 hours.

Strikingly, a very strong down-regulation of the THBS1 gene was observed after PMA/ionomycin stimulation for 24 hours with a reduced expression of 66 fold change by comparison to mock-stimulation (Table 3). This gene is also down-regulated in porcine aortic endothelial cells treated with PMA [50] and in bovine PBMCs stimulated with Concanavalin A [22]. Interestingly, THBS1 down-regulation has been shown to become stronger with time after Concanavalin A stimulation in bovine PBMCs [22], suggesting a persistent role of this gene repression during immune activation and a delayed response or no response for returning to pre-induction levels. THBS1 encodes an adhesive glycoprotein that mediates cell-to-cell and cell-to-matrix interactions. THBS1 has been described with many diverse functions that may relate to its structure and consequently to its ability to bind to matrix proteins, cell surface receptors or other molecules including cytokines [51]. THBS1 is known as a potent natural inhibitor of angiogenesis and endothelial cell migration [52]. THBS1 has been shown to be regulated by DNA methylation and to be a target for a transcription repression induced by the protein arginine methyltransferase 6 (PRMT6). Our work suggests that the strong repression of THBS1 observed in pig PBMCs may be due to methylation and that the PRMT6 gene may have a role in this repression. Interestingly, CD47, which encodes a membrane protein that is a receptor for the C-terminal cell binding domain of THBS1 [53] was also found slightly down-regulated (fold change of -1.4). Recent findings suggest that THBS1 contributes to the vascular system regulation by acting via its receptor CD47 to inhibit nitric oxide signaling [54]. Our findings suggest a major role of THBS1 repression in T/B cell activation upon stimulation with PMA/ionomycin, by enhancing the ability of cells to proliferate and migrate. Whether this role is connected to CD47 or to other receptors has to be further investigated.

Our study confirms an up-regulation of the pro-inflammatory cytokine IL8 but has not found an over-expression of IFNG as previously reported by Ledger *et al. *[25].

### Down-regulation of MHC mediated antigen presentation pathways after both stimulations

A strong down-regulation of MHC class II or MHC class I and II molecules was observed after LPS or PMA/ionomycin stimulation, respectively. Classical class II molecules are involved in antigen presentation to CD4+ T cells whereas classical class I genes have a double function of antigen presentation to CD8+ T cells and regulation of natural killer cell cytotoxicity by interacting with NK receptors such as NKG2D [55]. In pig, down-regulation of MHC genes has been reported in vivo in the spleen of animals infected by *H. parasuis *[9] and in vitro in PK15 cells infected by the pseudorabies virus [10] and in PBMCs stimulated with PMA/ionomycin [25]. In human, such a repression has also been reported in PBMCs infected by bacterial LPS and diverse killed bacteria [33]. Our results show that the repression program includes classical (DR and DQ series) and non classical (DM series) SLA class II genes after LPS and PMA/ionomycin stimulation as reported in human PBMCs. In addition, classical class I genes corresponding to SLA-3 and likely to SLA-1 and SLA-2 are also repressed together with the non classical genes SLA-6 and SLA-7 [56] that map to the SLA complex on chromosome 7 and CD1 that maps to chromosome 4 thus outside of the MHC locus. Strikingly, in our study, after PMA/ionomycin stimulation, biological networks connect the down-regulation of MHC class I molecules to a significant increase in transcription of numerous heat shock proteins known to act as chaperones as well as in transcription of all genes involved in the cascade of peptide processing before loading to the MHC molecule binding groove.

Induction of MHC class I expression is mainly transcriptional and promoters of class I genes contain IFN-stimulated response elements (ISRE) that bind factors of the IFN regulatory factor (IRF) family. Therefore, expression of IRFs influences transcription of class I genes. In our study, IRF1 and IRF8 are found up-regulated after PMA/ionomycin stimulation in contrast to IRF2 and IRF5 that are repressed. IRF8-mediated inhibition of antigen presentation by dendritic cells in the tumor microenvironment has been described in human [57] but our results are not in concordance with a possible role of IRF8 in MHC class I repression since the repression in peptide presentation by class I molecules was linked with a down-regulation of IRF8 together with a down-regulation of the peptide processing cascade [57]. In contrast, the down-regulation of IRF1 is in agreement with a possible role of this gene in inhibiting transcription of MHC class I genes [58].

### Comparison of transcriptomic signatures specific to LPS and PMA/ionomycin stimulations

In this study, about ten times more genes are found differentially expressed after PMA/ionomycin than after LPS stimulation. This might be related to the fact that LPS targets monocytes and macrophages [59] expressing CD14 and that PMA/ionomycin have a much wider spectrum of target cells. However, it cannot be ruled out that the significant difference in the number of differentially expressed genes according to stimulation is due to variations in the dynamics of the response. The onset of response may occur much earlier for LPS than for PMA/ionomycin. As a counterpart, the return to basal levels of gene transcription may also occur earlier after LPS stimulation, providing a possible hypothesis for a reduced number of differentially expressed genes after 24 hours stimulation. Additional studies are required to specifically address this question.

Specific and common features in transcriptome modifications were identified for both stimulations at 24 hours. Strikingly, the most significant similarly regulated genes after both stimulations are found down-regulated and many specific genes appear to be up-regulated.

Hierarchical clustering of genes found differentially expressed in both stimulation conditions provided a clear image of genes that were regulated either in the same direction or in opposite directions according to stimulation. In that respect, clusters C2, C4 and C7 (Figure 2) are the most informative to compare signatures and target possible markers that might be regulated in opposite directions according to stimulation. THBS1 (cluster C2), SAA1, chemokines CCL2, CXCL5 and CXCL6 (cluster C4) as well as IL1 receptor, immunoglobulins and LTB (cluster C7) provide a limited subset of genes that are specifically up-regulated after LPS stimulation and down-regulated after PMA/ionomycin stimulation. Similarly, cluster C7 including genes such as the chemokine CXCL10 and IRF8 provides a reservoir of genes specifically up-regulated after PMA/ionomycin stimulation and down-regulated after LPS stimulation.

### Role of non-coding transcripts

After LPS and PMA/ionomycin stimulation, quite a high number of probes corresponding to annotated transcripts in the anti-sense orientation are expressed. It is likely that the expressed antisense probes correspond to either new non annotated transcripts or to antisense transcripts from annotated genes. Interestingly, few of the anti-sense probes are differentially expressed after PMA/ionomycin stimulation suggesting a role in immune response activation that has to be further explored. These preliminary results on the expression of non-coding transcripts mapping to the SLA complex corresponds to a pilot study that would be worth extending to the whole genome.

## Conclusions

We have designed a long-oligonucleotide set (SLA-RI) comprising all the genes and pseudogenes annotated for the SLA complex as well as immune response genes outside the SLA complex and produced a generic array (SLA-RI/NRSP8-13K chip) enriched in immunity genes. We have assessed the relevance of this DNA chip by investigating the response of porcine PBMCs to two distinct stimuli LPS and PMA/ionomycin. Ours results reveal common as well as specific gene regulations according to stimulation, confirming some data already reported and providing new insights on the immune response in pig.

## Methods

### Probe selection and oligonucleotide design of the SLA-RI oligonucleotide set

To prepare the 816 probes targeting the SLA complex, all the annotated genes, pseudogenes and putative transcription variants were retrieved from the VEGA database [60]. Oligonucleotides were designed on both DNA strands (see Additional file 1: SLA_RI_Table_S1.xls). To select genes involved in immune response but located outside the SLA complex, a list was drawn up from the Porcine Immunology and Nutrition (PIN) database [61], the human Immunogenetic Related Information Source (IRIS) [62], the immune system pathway in KEGG [19], and immunology microarray resources, such as ARK-Genomics S. scrofa Immune Array 3 K v1.0 [14], the Affymetrix GeneChip^® ^Human Immune and Inflammation 9 K SNP Kit, Oligo GEArray^® ^Human Autoimmune and Inflammatory Response Microarray, Oligo GEArray^® ^Human Hematology/Immunology Microarray, Oligo GEArray^® ^Human Innate and Adaptive Immune Responses Microarray, Oligo GEArray^® ^Human Inflammatory Cytokines & Receptors Microarray, the PIQOR™ Immunology Microarray for human, and the PIQOR™ Immunology Microarray for mouse. Pig sequences were retrieved by GeneID and RefSeq search or by analysis for sequence similarity by BLAST (see Additional file 1: SLA_RI_Table_S1.xls). In cases where no pig sequence could be identified, a human sequence was used for the oligonucleotide design. Thus, the gene list comprises 2832 pig sequences and 125 human sequences and the final set consists of 2957 oligonucleotides. GO annotations of the probes were retrieved using the corresponding human RefSeq IDs [63,64]. Oligonucleotides were all designed and synthesized by Operon Company.

### Design and production of the SLA-RI/NRSP8-13K chip

The SLA-RI/NRSP8-13K chip was designed by combining the SLA-RI set with the NRSP8-13K set, which was purchased from the Operon Company. Oligonucleotides were resuspended in 0.5× Pronto! Universal Spotting Solution (Corning, USA) at a final concentration of 20 pmol/μL and printed on Corning UltraGAPS slides using a Chipwriter (Virtek, Canada) with 48 microspotting pins (SMP3, TeleChem International, Inc. USA). The Lucidea Universal ScoreCard control samples (GE Healthcare, USA) and SpotReport^® ^Alien^® ^cDNA Array Validation System control samples (Stratagene, USA) were both spotted in four replicates. After spotting, slides were air-dried and DNA was UV-fixed (600 mJ). Slides were stored in dry atmosphere before use. All information on SLA-RI/NRSP8-13 microarray platform has been submitted to the Gene Expression Omnibus (GEO) repository and the accession number is GPL7151. The DNA chips were produced by the French National platform CRB GADIE [65] and can be purchased upon request.

### Cell isolation and stimulation

PBMCs from seven Large White male pigs (~50 kg) were isolated by Ficoll-Hypaque density gradient centrifugation at room temperature. The PBMCs were cultured in RPMI 1640 medium (BioWhittaker, Belgium) supplemented with 10% heat-inactivated FBS (fetal bovine serum) (QB perbio, UK), 2 mmol/L L-glutamine, 100 U/mL penicillin and 100 mg/mL streptomycin. In our experimental conditions, 5 × 10^6 ^cells were incubated for 24 hours in culture medium supplemented with 1 μg/mL LPS from *E. coli *O111:B4 (Sigma, France) or a mixture of PMA (Sigma, France) at 10 ng/mL and ionomycin (Sigma, France) at 1 μg/mL. For mock-stimulation, cells were maintained in the culture medium for 24 hours. PBMCs were further centrifuged for 10 min at 4000 rpm and harvested for RNA extraction. Supernatants were frozen at -20°C for cytokine quantification by ELISA tests.

### RNA isolation and quality control

Total RNA was extracted from cells using the RNeasy Midi Kit (Qiagen, USA) and purified by on-column digestion of DNA with DNase I as recommended by the manufacturer (Qiagen, USA) to eliminate residual genomic DNA. RNA concentration was determined by Nanodrop quantification (Thermo Fisher Scientific Inc., USA). RNA quality was checked on an Agilent 2100 Bioanalyzer (Agilent Technologies, Germany). RNAs with a RIN score between 8 and 10 were labeled and used for microarray and qRT-PCR experiments. All RNAs were diluted to a final concentration of 1 μg/μL and stored at -80°C.

### RNA labelling, microarray hybridisation and signal quantification

For labelling, 5 μg of total RNA were reverse-transcribed and directly labelled by Cy3 or Cy5 using the ChipShot™ Direct Labeling System (Promega, USA). The CyDye-labelled cDNAs were purified using ChipShot™ Membrane Clean-Up System (Promega, USA). The absorbance at 260, 550 and 650 nm of CyDye-labelled cDNAs was measured by Nanodrop (Thermo Fisher Scientific Inc., USA). Frequency of incorporation (FOI) and labelling efficiency were checked by referring to standards provided by Labeled cDNA Calculator [66] (Promega, USA). The CyDye-labelled cDNAs were dried by vacuum centrifugation and resuspended at a final concentration of 2.5 pmol/μL in cDNA/long-oligonucleotide hybridization buffer. A dye swap hybridization scheme was designed to compare gene expression between mock-stimulated PBMCs and PBMCs stimulated by either LPS or a mixture of PMA and ionomycin. Each pig/condition RNA was labelled with Cy3 and Cy5 (Additional file 10: SLA_RI_Figure_S10.png). A total of 28 SLA-RI/NRSP8-13K chips were used in our study. Chip hybridization was performed using the Corning hybridization system. Prior to hybridization, the slides were treated with the background-reducing Pronto! Pre-Soak System (Corning, France) and then prehybridized using the Corning "Pronto! Universal Hybridization Solutions and Kits" (Product #40026). Hybridizations were carried out for 16 hours at 42°C in light-protected sealed Corning Hybridization Cassettes (Corning, Product #2551) placed in a water bath. The slides were washed according to the recommended protocol (Corning, Product #40026) and dried by centrifugation at 1600 rpm for 2 min. Slides were scanned using a GenePix™ 4000B array scanner (MDS Inc., Canada) and then array images were processed with the GenePix™ Pro software V6.0 (MDS Inc., Canada) to align spots, to integrate ID data files and to export reports of spot intensity data.

All the results were stored in the BioArray Software Environment (BASE) managed by SIGENAE [67]. The microarray data have been submitted to the GEO and received the accession number GSE17320.

### Microarray data statistical analysis

To identify any significant differential expression, the microarray data were analyzed using Limma (Linear Models for Microarray Data) [68] from the Bioconductor open-source project running under R [69,70]. After data pre-processing using within-array global loess normalization, the empirical eBayes method in Limma, which computes moderated t-statistics, moderated F-statistics, and log-odds of differential expression, was applied to identify the significance of differential expression in each culture condition. Adjustment for multiple testing was carried out using the false discovery rate (FDR) method [71] in Limma. Significant changes in gene expression were limited to p < 0.05. Hierarchical clustering analysis (HCL) was performed for gene classification [72] using the TMeV software [73].

### Significant functions and gene network analysis

The differentially expressed genes were analyzed using the IPA software (Ingenuity Systems, USA). Genes with known human locus IDs with corresponding differential expression values were uploaded into the software. Each human locus ID was mapped to its corresponding gene object in the Ingenuity Pathways Knowledge Base. Gene networks were algorithmically generated based on their connectivity and assigned a score. Ingenuity Pathways Analysis calculates a significance score for each network. The score is calculated using a p-value calculation for each network, and is displayed as the negative base-ten logarithm of that p-value. It indicates the likelihood that the assembly of a set of focus genes in a network may be explained by random chance alone. In this study, the cut-off significant score was set at 5, which means that a network score of 5 would only have approximately a 10^-5 ^chance of occurring randomly. The KEGG biology pathway information for differentially expressed genes was queried by ArrayTrack [74,75] using human locus IDs. The interconnectedness information was manually extracted from the KEGG pathways, and for simplicity a line connecting two KEGG pathways was used to represent these interactions. The interaction map was created using CytoScape software [76] to generate a framework of the interactions of the KEGG biological pathways.

### Quantitative real time RT-PCR (qRT-PCR)

Two μg of DNaseI-treated total RNAs were reverse-transcribed using Superscript II enzyme with Oligo(dT) primers. The cDNAs were quantified using a 2100 Bioanalyzer (Agilent Technologies, Germany) and diluted to a working concentration of 4 ng/μL. Duplicate reactions were performed in a final volume of 20 μL with 20 ng cDNA, 300 nM primers and SYBR Green PCR Master Mix (Applied Biosystem, USA), using an ABI PRISM 7900 HT sequence detection system (Applied Biosystem, USA). Primers were chosen either with the Primer Express Software or manually. The gene B2M was chosen as the internal reference gene and the 2^-ΔΔCt ^method was used to calculate the fold change in gene expression [77].

### ELISA test validation

For protein validation by ELISA tests, supernatants of mock-stimulated and stimulated PBMCs from the seven animals used for transcriptome analysis were tested. This means that supernatants for ELISA tests and PBMCs for RNA extraction and transcriptome analysis were collected at the same time from the same culture plates. The concentrations of IL8, IL12, IL1B and TNFA proteins were determined using commercially available ELISA kits (DuoSet, R&D Systems, USA), according to the manufacturer's instructions. Results were reported as the mean values of duplicate ELISA wells.

### FACS analysis

The anti-porcine MHC Class I monoclonal antibody PT85A (VMRD Inc., USA) and the anti-porcine MHC Class II monoclonal antibody MSA3 (VMRD Inc., USA) were used for FACS analysis. The monoclonal antibody HOPC-1 (IgG2a, Beckman Coulter, USA) was used as a control antibody for isotype. PE-conjugated goat antibodies to mouse IgG2a (Southern Biotech, USA) were used as a secondary antibody.

PBMCs from seven other Large White male pigs were stimulated and mock-stimulated in the same conditions as for microarray analysis. After centrifugation at 1500 rpm for 20 min at 4°C, cells were resuspended and incubated in pig serum for 25 min at 4°C. Cells were washed in PBS (BioWhittaker, Belgium) and incubated with 50 μL of diluted primary antibody (1/200 in FACS buffer) for 25 min at 4°C, then washed again and incubated with 50 μL of diluted PE-conjugated goat antimouse IgG2a (1/600 in FACS buffer) for 25 min at 4°C in light-protected chambers. After a final wash in PBS, PBMCs were fixed in BD CellFix (BD Biosciences, USA) solution and analyzed using a FACS Calibur flow cytometer (BD Biosciences, USA).

## Competing interests

The authors declare that they have no competing interests.

## Authors' contributions

YG designed the SLA-RI set and participated in working out the experimental design. He performed all RNA extractions, chip hybridizations, transcriptome analyses, qRT-PCR experiments, ELISA tests, FACS analyses and drafted the manuscript. LF participated in working out the experimental design, transcriptome analysis and FACS analysis. JL produced the SLA-RI/NRSP8-13K chip and carried out the quality controls together with DE. DE designed the chip spotting. ZH mapped the SLA-RI set on pig maps and included this oligonucleotide set in the animal QTL database. AT participated in ELISA tests. GL participated in the qRT-PCR experiments. FL and IPO contributed to the SLA-RI set design. CRG coordinated the whole study, contributed to the SLA-RI set design, the experimental design, the analysis and interpretation of the results and corrected the manuscript. All authors have read and approved the final manuscript.

## Supplementary Material

Additional file 1 **Detailed information of probes in the Porcine SLA-RI oligonucleotide set**. The file SLA_RI_Table_S1.xls is an excel file, which contains the detailed information and GO annotation information for the genes or probes included in the SLA-RI set. Click here for file

Additional file 2 **List of KEGG pathways represented in the SLA_RI set**. The file SLA_RI_Table_S2.doc is a word file, which contains the KEGG pathways represented in the SLA-RI. Click here for file

Additional file 3 **List of differentially expressed probes in opposite directions according to stimulation**. The file SLA_RI_Table_S3.doc is a word file, which contains the gene list of differentially expressed probes in opposite directions according to stimulation from hierarchical clustering (HCL) analysis. Click here for file

Additional file 4 **Hierarchical clustering of the 316 probes**. The file SLA_RI_Figure_S4.png is a portable network graphics file, which shows hierarchical clustering of the 316 probes that were found differentially expressed after LPS and PMA/ionomycin stimulations. Click here for file

Additional file 5 **LPS-related gene networks**. The file SLA_RI_Table_S5.xls is an excel file, which contains the network list obtained for LPS-related gene analysis. Click here for file

Additional file 6 **Details of KEGG biological pathways for genes differentially expressed with LPS or PMA/ionomycin stimulation**. The file SLA_RI_Table_S6.doc is a word file, which contains the detailed information of KEGG pathways for genes differentially expressed with LPS or PMA/ionomycin stimulation. Click here for file

Additional file 7 **PMA/ionomycin-related gene networks**. The file SLA_RI_Table_S7.xls is an excel file, which contains the network list obtained for PMA/ionomycin-related gene analysis. Click here for file

Additional file 8 **Comparison of fold change of gene expression level between microarray and qRT-PCR experiments**. The file SLA_RI_Table_S8.doc is a word file, which contains comparison results between microarray and qRT-PCR experiments. Click here for file

Additional file 9 **Detailed information of differentially expressed genes after LPS or PMA/ionomycin stimulation**. The file SLA_RI_Table_S9.xls is an excel file which contains two sheets. The "LPS" sheet contains the detailed information on differentially expressed genes after LPS stimulation, and the "PMA_ionomycin" sheet contains the detailed information on differentially expressed genes after PMA/ionomycin stimulation. Click here for file

Additional file 10 **Hybridization design**. The file SLA_RI_Figure_S10.png is a portable network graphics file, which shows the hybridization design used in this study to investigate the differentially expressed genes after LPS and PMA/ionomycin stimulations. Each arrow represents one microarray with a reversed labeling of cDNAs by Cy3 or Cy5. Arrow heads represent Cy5 and arrows point in the Cy3 to Cy5 direction. Click here for file
