# Supplementary material for: Transcriptome analysis of porcine PBMCs after in vitro stimulation by LPS or PMA/ionomycin using an expression array targeting the pig immune response
Source: BMC Genomics. 2010 May 11;11:292. doi: 10.1186/1471-2164-11-292 (PMC2881026; doi:10.1186/1471-2164-11-292)
Supplement: Additional file 2 — List of KEGG pathways represented in the SLA_RI set. The file SLA_RI_Table_S2.doc is a word file, which contains the KEGG pathways represented in the SLA-RI. [file 1471-2164-11-292-S2.DOC]

**Table S2.** List of KEGG pathways represented in the SLA_RI set

| KEGG pathway | Catalog | Number of genes | Fisher P value |
| --- | --- | --- | --- |
| Adherens junction | Cell Communication | 53 | 0 |
| Focal adhesion | Cell Communication | 152 | 0 |
| Tight junction | Cell Communication | 58 | 0.000207 |
| Apoptosis | Cell Growth and Death | 67 | 0 |
| Regulation of actin cytoskeleton | Cell Motility | 106 | 0.00000003 |
| Axon guidance | Development | 62 | 0.00015761 |
| Dorso-ventral axis formation | Development | 16 | 0.00700124 |
| Adipocytokine signaling pathway | Endocrine System | 44 | 0.00000071 |
| GnRH signaling pathway | Endocrine System | 50 | 0.00009691 |
| Insulin signaling pathway | Endocrine System | 59 | 0.00511879 |
| B cell receptor signaling pathway | Immune System | 57 | 0 |
| Complement and coagulation cascades | Immune System | 68 | 0 |
| Fc epsilon RI signaling pathway | Immune System | 63 | 0 |
| Hematopoietic cell lineage | Immune System | 83 | 0 |
| Leukocyte transendothelial migration | Immune System | 94 | 0 |
| Natural killer cell mediated cytotoxicity | Immune System | 94 | 0 |
| T cell receptor signaling pathway | Immune System | 81 | 0 |
| Toll-like receptor signaling pathway | Immune System | 78 | 0 |
| Antigen processing and presentation | Immune System | 46 | 0.00001102 |
| ErbB signaling pathway | Signal Transduction | 64 | 0 |
| Jak-STAT signaling pathway | Signal Transduction | 122 | 0 |
| MAPK signaling pathway | Signal Transduction | 130 | 0 |
| VEGF signaling pathway | Signal Transduction | 51 | 0 |
| TGF-beta signaling pathway | Signal Transduction | 52 | 0.00000004 |
| mTOR signaling pathway | Signal Transduction | 26 | 0.00121865 |
| Cell adhesion molecules (CAMs) | Signaling Molecules and Interaction | 117 | 0 |
| Cytokine-cytokine receptor interaction | Signaling Molecules and Interaction | 223 | 0 |
| ECM-receptor interaction | Signaling Molecules and Interaction | 71 | 0 |
| Proteasome | Folding, Sorting and Degradation | 19 | 0.00000001 |
| Chronic myeloid leukemia | Cancers | 62 | 0 |
| Colorectal cancer | Cancers | 56 | 0 |
| Endometrial cancer | Cancers | 45 | 0 |
| Glioma | Cancers | 48 | 0 |
| Non-small cell lung cancer | Cancers | 43 | 0 |
| Pancreatic cancer | Cancers | 65 | 0 |
| Prostate cancer | Cancers | 68 | 0 |
| Renal cell carcinoma | Cancers | 47 | 0 |
| Small cell lung cancer | Cancers | 76 | 0 |
| Melanoma | Cancers | 47 | 0.00000001 |
| Thyroid cancer | Cancers | 21 | 0.00007267 |
| Epithelial cell signaling in Helicobacter pylori infection | Infectious Diseases | 41 | 0.00000281 |
| Pathogenic Escherichia coli infection - EHEC | Infectious Diseases | 26 | 0.0027082 |
| Pathogenic Escherichia coli infection - EPEC | Infectious Diseases | 26 | 0.0027082 |
| Type I diabetes mellitus | Metabolic Disorders | 26 | 0.00030337 |
| Type II diabetes mellitus | Metabolic Disorders | 23 | 0.00597799 |
| Neurodegenerative Disorders | Neurodegenerative Diseases | 24 | 0.00021755 |
| Prion disease | Neurodegenerative Diseases | 10 | 0.00146112 |
| Total |  | 2960 |  |
