# Supplementary material for: Transcriptome analysis of porcine PBMCs after in vitro stimulation by LPS or PMA/ionomycin using an expression array targeting the pig immune response
Source: BMC Genomics. 2010 May 11;11:292. doi: 10.1186/1471-2164-11-292 (PMC2881026; doi:10.1186/1471-2164-11-292)
Supplement: Additional file 3 — List of differentially expressed probes in opposite directions according to stimulation. The file SLA_RI_Table_S3.doc is a word file, which contains the gene list of differentially expressed probes in opposite directions according to stimulation from hierarchical clustering (HCL) analysis. [file 1471-2164-11-292-S3.DOC]

**Table S3.** List of differentially expressed probes in opposite directions according to stimulation

| Oligo | Gene | Transcript | LPS Stimulation | PMA/ionomycin Stimulation |
| --- | --- | --- | --- | --- |
| Susc10003065 | THBS1 | AY773342.1 | Up regulation | Down regulation |
| SS00008318 | THBS1 | TC180813 | Up regulation | Down regulation |
|  |  |  |  |  |
| Susc10001999 | IGKV4-5 | AF334741.1 | Up regulation | Down regulation |
| Susc10002812 | SAA1 | NM_001044552.1 | Up regulation | Down regulation |
| SS00009822 | CTAPIII | - | Up regulation | Down regulation |
| SS00009845 | NP276195 | NP276195 | Up regulation | Down regulation |
| Susc10003114 | IGKV1-1 | AF334738.1 | Up regulation | Down regulation |
| SS00010608 | TC181370 | TC181370 | Up regulation | Down regulation |
| SS00011159 | TC183233 | TC183233 | Up regulation | Down regulation |
| SS00013129 | TC181368 | TC181368 | Up regulation | Down regulation |
| Susc10000342 | IGKVG502 | AF334742.1 | Up regulation | Down regulation |
| Susc10001382 | MCP-1 | NM_214214.1 | Up regulation | Down regulation |
| Susc10001848 | IgA | U12594.1 | Up regulation | Down regulation |
| Susc10001817 | IgG2b | U03780.1 | Up regulation | Down regulation |
| Susc10002129 | IGLL1 | AK237264.1 | Up regulation | Down regulation |
| Susc10002292 | CCL23 | BW955702.1 | Up regulation | Down regulation |
| SS00007572 | TC169972 | TC169972 | Up regulation | Down regulation |
| Susc10002435 | IGKV4-3 | AF334740.1 | Up regulation | Down regulation |
| Susc10002713 | SERPINB2 | AY609801.1 | Up regulation | Down regulation |
| SS00009850 | NP276226 | NP276226 | Up regulation | Down regulation |
| Susc10003442 | CTAPIII | NM_213862.1 | Up regulation | Down regulation |
| SS00010181 | TC185393 | TC185393 | Up regulation | Down regulation |
| SS00010452 | TC181514 | TC181514 | Up regulation | Down regulation |
| SS00000612 | TC190926 | TC190926 | Up regulation | Down regulation |
| SS00000465 | TC181626 | TC181626 | Up regulation | Down regulation |
| SS00000663 | - |  | Up regulation | Down regulation |
| Susc10003568 | CCL2 | EF107669.1 | Up regulation | Down regulation |
| Susc10000030 | IGKV2-4 | AF334739.1 | Up regulation | Down regulation |
| Susc10000318 | CXCL5 | NM_213876.1 | Up regulation | Down regulation |
| SS00000977 | TC162938 | TC162938 | Up regulation | Down regulation |
| SS00000905 | TC163813 | TC163813 | Up regulation | Down regulation |
| SS00000842 | TC163286 | TC163286 | Up regulation | Down regulation |
| SS00001328 | TC162941 | TC162941 | Up regulation | Down regulation |
| SS00001329 | TC162942 | TC162942 | Up regulation | Down regulation |
| Susc10001012 | Igh-V | DQ886394.1 | Up regulation | Down regulation |
| Susc10000945 | IgG4 | U03782.1 | Up regulation | Down regulation |
| Susc10001738 | IL1R2 | DB819692.1 | Up regulation | Down regulation |
| Susc10003318 | IgM | AB205164.1 | Up regulation | Down regulation |
| SS00002453 | TC162977 | TC162977 | Up regulation | Down regulation |
| SS00005143 | TC163163 | TC163163 | Up regulation | Down regulation |
|  |  |  |  |  |
| Susc10000705 | AMICA1 | BP161558.1 | Down regulation | Up regulation |
| Susc10002317 | CXCL10 | NM_001008691.1 | Down regulation | Up regulation |
| Susc10000189 | IRF8 | DY434707.1 | Down regulation | Up regulation |
| SS00004202 | ETFB | TC163232 | Down regulation | Up regulation |
| SS00001624 | POLR1D | TC183130 | Down regulation | Up regulation |
| SS00008516 | EGR1 | TC186531 | Down regulation | Up regulation |
|  |  |  |  |  |
| SS00004915 | ANKZF1 | TC186364 | Down regulation | Up regulation |
| SS00003281 | TC163340 | TC163340 | Up regulation | Down regulation |
| Susc10000945 | IgG4 | U03782.1 | Up regulation | Down regulation |
| SS00003896 | TC181768 | TC181768 | Up regulation | Down regulation |
| SS00003981 | TC164436 | TC164436 | Up regulation | Down regulation |
| Susc10001490 | TGFBI | BP170635.1 | Up regulation | Down regulation |
| SS00001180 | TC162668 | TC162668 | Up regulation | Down regulation |
| SS00005129 | TC163956 | TC163956 | Up regulation | Down regulation |
| SS00001180 | TC162668 | TC162668 | Up regulation | Down regulation |
| SS00004115 | TC162962 | TC162962 | Up regulation | Down regulation |
| Susc10001527 | MS4A1 | AK236877.1 | Up regulation | Down regulation |
| Susc10001382 | MCP-1 | NM_214214.1 | Up regulation | Down regulation |
| SS00004292 | TC164126 | TC164126 | Up regulation | Down regulation |
| SS00004915 | TC186364 | TC186364 | Up regulation | Down regulation |
| SS00004283 | TC182502 | TC182502 | Up regulation | Down regulation |
| SS00003495 | TC182079 | TC182079 | Up regulation | Down regulation |
| SS00005143 | TC163163 | TC163163 | Up regulation | Down regulation |
| SS00004261 | TC163456 | TC163456 | Up regulation | Down regulation |
| Susc10001743 | NQO1 | AY610195.1 | Up regulation | Down regulation |
| SS00005446 | TC163680 | TC163680 | Up regulation | Down regulation |
| SS00005762 | TC181365 | TC181365 | Up regulation | Down regulation |
| SS00006169 | TC164291 | TC164291 | Up regulation | Down regulation |
| Susc10001945 | AEP | AK239674.1 | Up regulation | Down regulation |
| Susc10001613 | SLA-DQB | SLA-DQB | Up regulation | Down regulation |
| SS00005339 | TC166337 | TC166337 | Up regulation | Down regulation |
| Susc10002129 | IGLL1 | AK237264.1 | Up regulation | Down regulation |
