# Supplementary figures and images for: Transcriptome analysis of porcine PBMCs after in vitro stimulation by LPS or PMA/ionomycin using an expression array targeting the pig immune response
Source: BMC Genomics. 2010 May 11;11:292. doi: 10.1186/1471-2164-11-292 (PMC2881026; doi:10.1186/1471-2164-11-292)

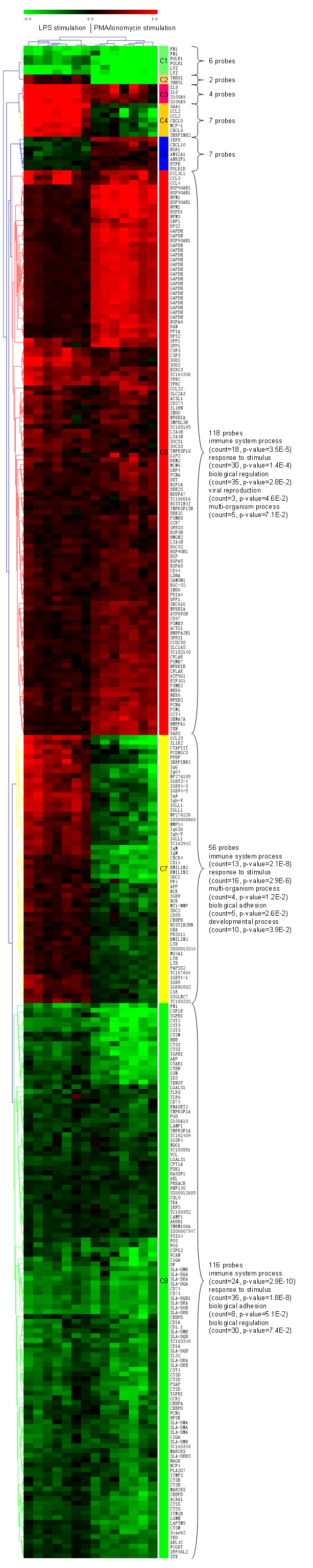

Supplement: Additional file 4 — Hierarchical clustering of the 316 probes. The file SLA_RI_Figure_S4.png is a portable network graphics file, which shows hierarchical clustering of the 316 probes that were found differentially expressed after LPS and PMA/ionomycin stimulations. [file 1471-2164-11-292-S4.PNG]

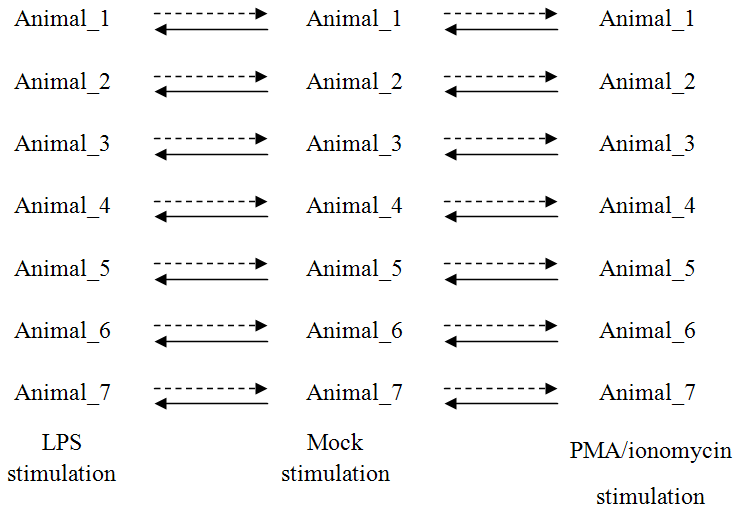

Supplement: Additional file 10 — Hybridization design. The file SLA_RI_Figure_S10.png is a portable network graphics file, which shows the hybridization design used in this study to investigate the differentially expressed genes after LPS and PMA/ionomycin stimulations. Each arrow represents one microarray with a reversed labeling of cDNAs by Cy3 or Cy5. Arrow heads represent Cy5 and arrows point in the Cy3 to Cy5 direction. [file 1471-2164-11-292-S10.PNG]
