# Supplementary material for: Transcriptome analysis of porcine PBMCs after in vitro stimulation by LPS or PMA/ionomycin using an expression array targeting the pig immune response
Source: BMC Genomics. 2010 May 11;11:292. doi: 10.1186/1471-2164-11-292 (PMC2881026; doi:10.1186/1471-2164-11-292)
Supplement: Additional file 6 — Details of KEGG biological pathways for genes differentially expressed with LPS or PMA/ionomycin stimulation. The file SLA_RI_Table_S6.doc is a word file, which contains the detailed information of KEGG pathways for genes differentially expressed with LPS or PMA/ionomycin stimulation. [file 1471-2164-11-292-S6.DOC]

**Table S6.** Detail of KEGG biological pathways for genes differentially expressed with LPS or PMA/ionomycin stimulation

| Condition | KEGG pathway | observed genes (Human) |
| --- | --- | --- |
| LPS Stimulation | Acute myeloid leukemia | CEBPA, NFKB2, PIK3CD, PIK3R5, PIM1 |
|  | Adipocytokine signaling pathway | ACSL1, CPT1A, NFKB2, NFKBIA, NFKBIB, SOCS3, TNFRSF1A |
|  | Alzheimer's disease | APP, C1QA, GAPDH, IL1B |
|  | Antigen processing and presentation | CD74, CTSB, CTSL1, CTSS, HLA-DMA, HLA-DMB, HLA-DOB, HLA-DQA1, HLA-DQB1, HLA-DRA, HLA-DRB1, HSP90AB1, HSPA5, LGMN, PSME2 |
|  | Apoptosis | BIRC3, CFLAR, IL1A, IL1B, IL1RAP, NFKB2, NFKBIA, PIK3CD, PIK3R5, PRKACB, TNFRSF1A |
|  | B cell receptor signaling pathway | FOS, IFITM1, LYN, NFKB2, NFKBIA, NFKBIB, PIK3CD, PIK3R5 |
|  | Bladder cancer | E2F1, IL8, MMP9, RASSF1, THBS1 |
|  | Cell adhesion molecules | ALCAM, CADM1, CD274, HLA-DMA, HLA-DMB, HLA-DOB, HLA-DQA1, HLA-DQB1, HLA-DRA, HLA-DRB1, SDC1, SDC2, VCAN |
|  | Complement and coagulation cascades | C1QA, C1R, C5AR1, CD55, CFD, PLAUR |
|  | Cytokine-cytokine receptor interaction | CCL22, CCL23, CCL2, CCL3, CCL4, CCL8, CCR2, CCR7, CSF1R, CSF3, CXCL10, CXCL2, CXCL3, CXCL5, CXCL6, CXCR4, IL10RB, IL1A, IL1B, IL1R2, IL1RAP, IL8, LTB, PF4, PPBP, TNFRSF13B, TNFRSF18, TNFRSF1A, TNFSF11 |
|  | Epithelial cell signaling in Helicobacter pylori infection | ATP6V0B, ATP6V1B2, IL8, LYN, NFKB2, NFKBIA |
|  | Hematopoietic cell lineage | CD14, CD1A, CD44, CD55, CSF1R, CSF3, IL1A, IL1B, IL1R2, MS4A1, HLA-DRA, HLA-DRB1, TFRC |
|  | Small cell lung cancer | BIRC3, E2F1, FN1, NFKB2, NFKBIA, PIK3CD, PIK3R5 |
|  | T cell receptor signaling pathway | FOS, NFKB2, NFKBIA, NFKBIB, PDK1, PIK3CD, PIK3R5 |
|  | Toll-like receptor signaling pathway | CCL3, CCL4, CD14, CXCL10, FOS, IL1B, IL8, IRF5, NFKB2, NFKBIA, PIK3CD, PIK3R5, SPP1, TLR6, TLR8 |
|  | Type I diabetes mellitus | HSPD1, IL1A, IL1B, HLA-DMA, HLA-DMB, HLA-DOB, HLA-DQA1, HLA-DQB1, HLA-DRA, HLA-DRB1 |
| PMA/ionomycin Stimulation | Acute myeloid leukemia | ARAF, CEBPA, EIF4EBP1, IKBKG, LEF1, MAP2K2, MAPK1, MAPK3, MYC, NFKB1, NFKB2, PIK3CG, PIK3R1, PIK3R2, PIM1, PIM2, PML, PPARD, RARA, SPI1, STAT3, STAT5A, TCF7 |
|  | Adherens junction | IQGAP1, LEF1, MAPK1, MAPK3, PTPN1, PTPRF, RAC1, RAC2, RHOA, SMAD4, SNAI1, TCF7, TGFBR1, TGFBR2, VCL |
|  | Adipocytokine signaling pathway | ACP1, ACTG1, ACTN1, CREBBP, CSNK2A1, CSNK2B, CTNNB1, CTNND1, EP300, FYN, ACSL1, ACSL4, ACSL5, ADIPOR1, ADIPOR2, CAMKK2, CPT1A, IKBKG, JAK1, JAK3, LEP, NFKB1, NFKB2, NFKBIA, NFKBIB, PCK2, PTPN11, RXRB, SLC2A1, SOCS3, STAT3, TNF, TNFRSF1A, TYK2 |
|  | Aminoacyl-tRNA biosynthesis | AARS, FARSA, FARSB, GARS, HARS, IARS2, KARS, LARS, RARS, SARS, TARS2, TARS, VARS, WARS, YARS |
|  | Antigen processing and presentation | CALR, CANX, CD4, CD74, CD8A, CD8B, CIITA, CTSB, CTSS, HLA-A, HLA-DMA, HLA-DMB, HLA-DOA, HLA-DOB, HLA-DQA1, HLA-DQB1, HLA-DRA, HLA-DRB1, HSP90AA1, HSP90AB1, HSPA1A, HSPA1B, HSPA1L, HSPA4, HSPA5, HSPA8, IFI30, IFNA5, KLRD1, LGMN, LTA, PDIA3, PSME1, PSME2, PSME3, TAP1, TAP2 |
|  | B cell receptor signaling pathway | BTK, CARD11, CD19, CD72, CD79A, CD79B, CD81, FCGR2B, FOS, IKBKG, INPP5D, JUN, MALT1, NFAT5, NFATC2, NFATC3, NFKB1, NFKB2, NFKBIA, NFKBIB, PIK3CG, PIK3R1, PIK3R2, PRKCB1, RAC1, RAC2, SYK, VAV3 |
|  | Biotin metabolism | BTD, SPCS1, SPCS3 |
|  | Cell adhesion molecules (CAMs) | CADM1, CD274, CD276, CD28, CD40, CD40LG, CD4, CD6, CD86, CD8A, CD8B, CD99, CLDN10, CLDN1, CLDN5, HLA-A, HLA-DMA, HLA-DMB, HLA-DOA, HLA-DOB, HLA-DQA1, HLA-DQB1, HLA-DRA, HLA-DRB1, ICAM1, ICAM3, ICOS, ICOSLG, ITGA4, ITGA6, ITGAV, ITGB2, ITGB7, NFASC, NLGN2, NRXN1, OCLN, PECAM1, PTPRC, PTPRF, SDC1, SDC2, SDC3, SELL, SELPLG, SIGLEC1, VCAN |
|  | Cell cycle | ANAPC11, ANAPC5, ATM, BUB3, CCND2, CCND3, CDC20, CDC45L, CDK2, CDK4, CDK6, CDKN1A, CDKN1B, CDKN2B, CDKN2D, CREBBP, EP300, ESPL1, GADD45A, HDAC1, HDAC2, MAD2L1, MAD2L2, MCM2, MCM3, MCM4, MCM5, MCM6, PCNA, RBL2, SKP1, SMAD4, SMC1A, TFDP1, TGFB1, TGFB2, YWHAE, YWHAG, YWHAH, YWHAQ, YWHAZ |
|  | Chronic myeloid leukemia | ARAF, BCL2L1, CBLB, CDK4, CDK6, CDKN1A, CDKN1B, GAB2, HDAC1, HDAC2, IKBKG, MAP2K2, MAPK1, MAPK3, MYC, NFKB1, NFKB2, NFKBIA, PIK3CG, PIK3R1, PIK3R2, PTPN11, SHC1, SMAD4, STAT5A, STAT5B, TGFB1, TGFB2, TGFBR1, TGFBR2 |
|  | Citrate cycle (TCA cycle) | CS, DLD, FH, IDH3G, MDH1, MDH2, PCK2, SDHA, SDHB, SDHD, SUCLA2, SUCLG1, SUCLG2 |
|  | Cytokine-cytokine receptor interaction | ACVR2A, AMH, CCL20, CCL22, CCL23, CCL2, CCL3, CCL4, CCL7, CCR1, CCR2, CCR4, CCR6, CCR7, CD40, CD40LG, CSF1, CSF1R, CSF2, CSF3, CSF3R, CX3CR1, CXCL10, CXCL16, CXCL5, CXCL6, CXCR4, FAS, IFNA5, IFNAR1, IFNAR2, IFNGR1, IFNGR2, IL10RA, IL12B, IL12RB2, IL13RA1, IL15RA, IL17B, IL17RA, IL18, IL1R2, IL21R, IL29, IL2, IL2RA, IL2RG, IL6, IL6R, IL7R, IL8, IL8RA, INHBA, LEP, LIF, LTA, LTB, LTBR, PDGFB, PF4, PPBP, TGFB1, TGFB2, TGFBR1, TGFBR2, TNF, TNFRSF13B, TNFRSF18, TNFRSF19, TNFRSF1A, TNFRSF21, TNFRSF6B, TNFRSF9, XCL1 |
|  | Fatty acid elongation in mitochondria | ECHS1, HADHA, HADHB, HSD17B10, PPT1, PPT2 |
|  | Fc epsilon RI signaling pathway | BTK, CSF2, FCER1G, FYN, GAB2, INPP5D, LCP2, MAP2K2, MAP2K3, MAPK1, MAPK3, PDK1, PIK3CG, PIK3R1, PIK3R2, PLA2G1B, PLCG1, PRKCA, PRKCB1, PRKCD, RAC1, RAC2, SYK, TNF, VAV3 |
|  | Glycolysis / Gluconeogenesis | ACSS2, ADH5, AKR1A1, ALDH2, ALDH9A1, ALDOA, DLD, ENO1, GAPDH, GPI, HK1, LDHA, LDHB, PDHA1, PDHB, PFKL, PFKP, PGAM1, PGAM2, PGK1, PGM3, PKM2, TPI1 |
|  | Glycosphingolipid biosynthesis - globoseries | A4GALT, HEXA, HEXB, NAGA, ST3GAL1, ST3GAL2, ST8SIA1 |
|  | Hematopoietic cell lineage | CD14, CD19, CD1A, CD24, CD33, CD37, CD38, CD3E, CD44, CD4, CD55, CD59, CD5, CD7, CD8A, CD8B, CSF1, CSF1R, CSF2, CSF3, CSF3R, GP9, HLA-DRA, HLA-DRB1, IL1R2, IL2RA, IL6, IL6R, IL7R, ITGA1, ITGA2, ITGA4, ITGA5, ITGA6, ITGB3, MS4A1, TFRC, TNF |
|  | Jak-STAT signaling pathway | BCL2L1, CBLB, CCND2, CCND3, CISH, CREBBP, CSF2, CSF3, CSF3R, EP300, IFNA5, IFNAR1, IFNAR2, IFNGR1, IFNGR2, IL10RA, IL12B, IL12RB2, IL13RA1, IL15RA, IL21R, IL29, IL2, IL2RA, IL2RG, IL6, IL6R, IL7R, JAK1, JAK3, LEP, LIF, MYC, PIK3CG, PIK3R1, PIK3R2, PIM1, PTPN11, SOCS1, SOCS3, STAT1, STAT3, STAT5A, STAT5B, STAT6, TYK2 |
|  | Leukocyte transendothelial migration | ACTG1, ACTN1, ARHGAP5, CD99, CLDN10, CLDN1, CLDN5, CTNNB1, CTNND1, CXCR4, CYBA, CYBB, GNAI2, ICAM1, ITGA4, ITGB2, NCF1, NCF2, NCF4, OCLN, PECAM1, PIK3CG, PIK3R1, PIK3R2, PLCG1, PRKCA, PRKCB1, PTK2B, PTPN11, RAC1, RAC2, RAP1A, RAP1B, RASSF5, RHOA, THY1, TXK, VAV3, VCL |
|  | Natural killer cell mediated cytotoxicity | ARAF, BID, CASP3, CD247, CD48, CSF2, FAS, FCER1G, FCGR3B, FYN, GZMB, HLA-A, ICAM1, IFNA5, IFNAR1, IFNAR2, IFNGR1, IFNGR2, ITGB2, KLRD1, LCP2, MAP2K2, MAPK1, MAPK3, NFAT5, NFATC2, NFATC3, PAK1, PIK3CG, PIK3R1, PIK3R2, PLCG1, PRKCA, PRKCB1, PTK2B, PTPN11, RAC1, RAC2, SH2D1A, SHC1, SYK, TNF, TYROBP, VAV3 |
|  | Neurodegenerative Diseases | APOE, APP, BCL2L1, CASP1, CASP3, CREBBP, EP300, FBXW7, GAPDH, HSPA5, NR4A2, PARK7, PRNP, PSEN2, SETX, SOD1 |
|  | Oxidative phosphorylation | ATP5A1, ATP5B, ATP5C1, ATP5D, ATP5E, ATP5F1, ATP5G1, ATP5G2, ATP5G3, ATP5H, ATP5J2, ATP5J, ATP5L, ATP5O, ATP6V0B, ATP6V0C, ATP6V1A, ATP6V1E1, ATP6V1G1, COX10, COX5A, COX5B, COX6C, COX7A2, COX7C, CYC1, NDUFA10, NDUFA12, NDUFA13, NDUFA2, NDUFA4, NDUFA4L2, NDUFA7, NDUFA8, NDUFA9, NDUFB2, NDUFB3, NDUFB4, NDUFB5, NDUFB6, NDUFB7, NDUFB9, NDUFC1, NDUFC2, NDUFS1, NDUFS2, NDUFS5, NDUFS7, NDUFS8, NDUFV1, NDUFV2, PPA1, SDHA, SDHB, SDHD, TCIRG1, UCRC, UQCR, UQCRB, UQCRC1, UQCRC2, UQCRFS1, UQCRQ |
|  | Pathogenic Escherichia coli infection - EHEC | ACTG1, ARPC5, ARPC5L, CD14, CLDN1, CTNNB1, CTTN, FYN, HCLS1, NCL, OCLN, PRKCA, RHOA, TLR4, TUBA1A, TUBA1B, TUBA1C, TUBB2A, TUBB, YWHAQ, YWHAZ |
|  | Pathogenic Escherichia coli infection - EPEC | ACTG1, ARPC5, ARPC5L, CD14, CLDN1, CTNNB1, CTTN, FYN, HCLS1, NCL, OCLN, PRKCA, RHOA, TLR4, TUBA1A, TUBA1B, TUBA1C, TUBB2A, TUBB, YWHAQ, YWHAZ |
|  | Phenylalanine, tyrosine and tryptophan biosynthesis | FARSA, FARSB, GOT1, GOT2, YARS |
|  | Prion disease | HSPA5, HSPD1, IL6, LAMC1, NFE2L2, PRNP, RPSA, TNF |
|  | Propanoate metabolism | ACAT1, ACSS2, ALDH2, ALDH9A1, ECHS1, HADHA, LDHA, LDHB, MUT, PCCB, SUCLA2, SUCLG1, SUCLG2 |
|  | Proteasome | PSMA1, PSMA2, PSMA3, PSMA4, PSMA5, PSMA6, PSMA7, PSMB1, PSMB2, PSMB3, PSMB4, PSMB5, PSMB6, PSMB7, PSMC3, PSMD11, PSMD12, PSMD13, PSMD1, PSMD2, PSMD6 |
|  | Pyrimidine metabolism | CAD, CMPK1, DHODH, DTYMK, DUT, ENTPD4, ITPA, NME1, NME2, NT5C2, PNPT1, POLA2, POLD4, POLE3, POLR1A, POLR1C, POLR1D, POLR2E, POLR2G, POLR2H, POLR2K, RRM2, TK1, TYMS, UCK1, UCK2, UMPS, UPP1, UPRT, ZNRD1 |
|  | Pyruvate metabolism | ACAT1, ACSS2, AKR1B1, ALDH2, ALDH9A1, DLD, GRHPR, HAGHL, LDHA, LDHB, LDHD, MDH1, MDH2, PCK2, PDHA1, PDHB, PKM2 |
|  | Renal cell carcinoma | ARAF, CREBBP, EP300, ETS1, FH, HIF1A, JUN, MAP2K2, MAPK1, MAPK3, PAK1, PDGFB, PIK3CG, PIK3R1, PIK3R2, PTPN11, RAC1, RAP1A, RAP1B, RAPGEF1, SLC2A1, TCEB1, TGFB1, TGFB2 |
|  | Ribosome | C15orf15, FAU, LOC284064, RPL10A, RPL11, RPL12, RPL13, RPL14, RPL18A, RPL19, RPL23A, RPL24, RPL26, RPL27, RPL27A, RPL28, RPL29, RPL30, RPL31, RPL32, RPL34, RPL35A, RPL36A, RPL36AL, RPL37, RPL37A, RPL39, RPL3, RPL6, RPL7, RPL8, RPS10, RPS11, RPS12, RPS15A, RPS16, RPS18, RPS20, RPS21, RPS23, RPS24, RPS25, RPS26, RPS27, RPS28, RPS29, RPS2, RPS3, RPS3A, RPS5, RPS6, RPS7, RPS8, RPS9, RPSA |
|  | T cell receptor signaling pathway | CARD11, CBLB, CD247, CD28, CD3E, CD40LG, CD4, CD8A, CD8B, CDK4, CSF2, FOS, FYN, GRAP2, ICOS, IKBKG, IL2, JUN, LCP2, MALT1, NFAT5, NFATC2, NFATC3, NFKB1, NFKB2, NFKBIA, NFKBIB, PAK1, PDK1, PIK3CG, PIK3R1, PIK3R2, PLCG1, PTPRC, RASGRP1, RHOA, TNF, VAV3 |
|  | TGF-beta signaling pathway | ACVR2A, AMH, CDKN2B, CREBBP, E2F4, EP300, ID1, ID2, ID3, ID4, INHBA, MAPK1, MAPK3, MYC, PPP2CA, PPP2R1A, PPP2R1B, RBL2, RHOA, SKP1, SMAD4, SP1, TFDP1, TGFB1, TGFB2, TGFBR1, TGFBR2, THBS1, THBS2, TNF |
|  | Thyroid cancer | CTNNB1, LEF1, MAP2K2, MAPK1, MAPK3, MYC, NTRK1, PPARG, RXRB, TCF7, TPM3, TPR |
|  | Toll-like receptor signaling pathway | CCL3, CCL4, CD14, CD40, CD86, CXCL10, FOS, IFNA5, IFNAR1, IFNAR2, IKBKE, IKBKG, IL12B, IL6, IL8, IRF5, JUN, MAP2K2, MAP2K3, MAP3K7IP1, MAPK1, MAPK3, MYD88, NFKB1, NFKB2, NFKBIA, PIK3CG, PIK3R1, PIK3R2, RAC1, SPP1, STAT1, TBK1, TLR1, TLR4, TLR6, TLR8, TNF, TOLLIP, TRAF3 |
|  | Type I diabetes mellitus | CD28, CD86, FAS, GZMB, HLA-A, HLA-DMA, HLA-DMB, HLA-DOA, HLA-DOB, HLA-DQA1, HLA-DQB1, HLA-DRA, HLA-DRB1, HSPD1, IL12B, IL2, LTA, TNF |
