# Supplementary material for: Transcriptome analysis of porcine PBMCs after in vitro stimulation by LPS or PMA/ionomycin using an expression array targeting the pig immune response
Source: BMC Genomics. 2010 May 11;11:292. doi: 10.1186/1471-2164-11-292 (PMC2881026; doi:10.1186/1471-2164-11-292)
Supplement: Additional file 8 — Comparison of fold change of gene expression level between microarray and qRT-PCR experiments. The file SLA_RI_Table_S8.doc is a word file, which contains comparison results between microarray and qRT-PCR experiments. [file 1471-2164-11-292-S8.DOC]

**Table S8.** Comparison of fold change of gene expression level between microarray and qRT-PCR experiments

|  | LPS stimulation VS. mock stimulation | | PMA/ionomycin stimulation VS. mock stimulation | |
| --- | --- | --- | --- | --- |
|  | fold change | | fold change | |
|  | qPCR | Microarray | qPCR | Microarray |
| CD69 | 1.13 ^ND^ | 1.19 ^ND^ | 23.10 | 6.87 |
| CST2 | -4.96 | -2.68 | -35.26 | -5.82 |
| IL1A | 12.73 | 4.53 | 1.23 ^ND^ | 1.10 ^ND^ |
| PSMB9 | -1.06 ^ND^ | 1.02 ^ND^ | 3.10 | 2.43 |
| PSMB8 | 1.19 | 1.27 | 2.06 | 2.10 |
| LYZ | -41.36 | -8.59 | -2336.28 | -14.76 |
| PPIA | 1.08 | 1.27 | 11.47 | 4.32 |
| SLAIa | -1.34 | -1.04 ^ND^ | -1.80 | -1.33 |
| SLA-DQB | -3.20 | -2.34 | -10.27 | -2.62 |
| SLA-DRA | -2.22 | -2.50 | -4.66 | -2.83 |
| SLA-DRB1 | -3.25 | -2.28 | -6.92 | -3.12 |
| TAP1 | 1.37 | 1.28 | 2.41 | 1.85 |
| TAP2 | 1.12 ^ND^ | 1.16 ^ND^ | 1.52 | 1.46 |
| TNFRSF9 | -1.12 ^ND^ | 1.04 ^ND^ | 95.67 | 6.63 |

ND means the gene expression change with stimulation did not reach 0.05 significance in microarray or qRT-PCR analysis.
